# Fluorine-Free Ion Exchange Membranes for (Photo)electrochemical Applications

**DOI:** 10.1021/acspolymersau.5c00089

**Published:** 2025-10-20

**Authors:** Dzenna Zukova, Martin D. Hager, Felix H. Schacher, Roel van de Krol, Ulrich S. Schubert, Marco Favaro

**Affiliations:** † Institute for Solar Fuels, 28340 Helmholtz-Zentrum Berlin für Materialien und Energie GmbH, Hahn-Meitner-Platz 1, 14109 Berlin, Germany; ‡ Laboratory of Organic and Macromolecular Chemistry (IOMC), 9378 Friedrich Schiller University Jena, Humboldtstraße 10, 07743 Jena, Germany; § Helmholtz Institute for Polymers in Energy Applications Jena (HIPOLE Jena), Lessingstraße 12−14, 07743 Jena, Germany; ∥ Jena Center for Soft Matter (JCSM), Friedrich Schiller University Jena, Philosophenweg 7, 07743 Jena, Germany; ⊥ Center for Energy and Environmental Chemistry Jena (CEEC), Friedrich Schiller University Jena, Philosophenweg 7a, 07743 Jena, Germany; # Institut für Chemie, Technische Universität Berlin, Straße des 17. Juni 124, 10623 Berlin, Germany

**Keywords:** Photoelectrochemical devices, Solar fuels, Solar chemicals, Electrolyzers, Ion exchange membranes, Fluorine-free membranes, Sustainable membrane fabrication, Electrochemical impedance spectroscopy, Ambient pressure X-ray photoelectron spectroscopy, AI-driven materials discovery

## Abstract

The increasing global demand for sustainable energy solutions has driven significant advancements in photoelectrochemical (PEC) technologies, particularly for hydrogen production and biomass valorization. A key challenge for PEC cells is the selection of ion exchange membranes (IEMs) that ensure efficient product separation between anode and cathode half-cells while enabling efficient ion transport. Moreover, these membranes also need to show long-term stability. Traditionally, perfluorinated membranes such as Nafion have been widely used due to their high proton conductivity and chemical resilience. However, their high cost, environmental concerns, and the impending regulatory restrictions on per- and polyfluoroalkyl substances necessitate the development of fluorine-free alternatives. This review explores the latest advancements in fluorine-free IEMs for (photo)­electrochemical applications, highlighting their synthesis, physicochemical properties, appropriate characterization methods, and performance metrics. We discuss emerging materials that offer comparable ionic conductivity, durability, and operational efficiency while addressing recyclability and environmental impact. By assessing the potential of these next-generation membranes, we aim to provide insights into their role in advancing photo- and electrochemical systems toward a more sustainable and economically viable future.

## Graphical abstract

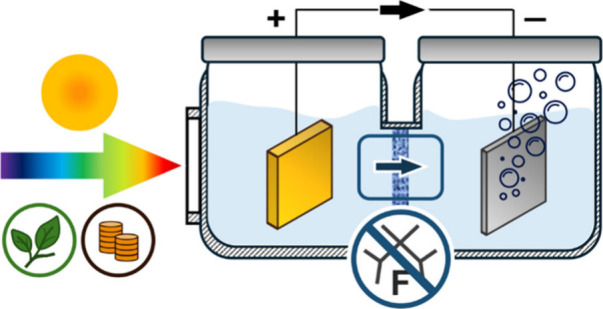

## 1 Introduction

With the onset of the industrial era, the emissions of CO_2_ gas have risen steadily, leading to irreversible consequences for climate change. A significant percentage of these emissions comes from using fossil fuel-derived energy sources such as oil and gas. The use of renewable energy sources (RESs) would significantly reduce the emissions of greenhouse gases (GHGs) and contribute to a sustainable development. Among various technologies, photoelectrochemical (PEC) devices constitute a promising technology in the transition toward sustainable energy solutions. The production of green hydrogen (H_2_) through water splitting, and more recently, the use of biomass-derived molecules^1–5^ as clean energy carriers powered by solar energy, are a primary focus in the development of PEC cells. A PEC cell integrates the process of electrolysis with sunlight as the energy source, utilizing semiconducting materials to generate photoexcited charge carriers that drive the electrochemical water-splitting reaction. The latter involves the decomposition of water (H_2_O) through the hydrogen evolution reaction (HER) at the cathodic side, producing H_2_, and the oxygen evolution reaction (OER) at the anodic side, producing oxygen (O_2_). Various photoelectrodes and catalysts can be utilized to perform the overall process.^6–11^

Despite many research efforts, the OER still faces significant kinetic limitations. Moreover, oxygen has a relatively low economic value, which makes its production less appealing.^12^ To address these challenges, the OER can be replaced with an alternative half-cell reaction to produce highly valuable chemical products. This “paired electrolysis” or “hybrid electrolysis”^13^ helps to overcome numerous issues and has advantages in terms of efficiency, making the overall process more economically viable.^14^ An example of an alternative anodic reaction is the oxidation of glycerol, the main byproduct of the transesterification of lipids to biodiesel (see Scheme 1).^15^ The Gibbs free energy for the glycerol oxidation reaction (GOR) coupled with HER is Δ*G* = +3.9 kJ·mol^–1^, whereas hydrogen production through direct water splitting has a much higher Gibbs free energy of Δ*G* = +237.2 kJ·mol^–1^. This indicates that GOR requires a significantly lower energy input than water splitting. Moreover, GOR as the anodic process in photoelectrolyzers lowers the kinetic overpotentials (and, thus, the operating cell voltage), while increasing at the same time the contact selectivity for holes.^1,16,17^ Thus, solar-driven biomass reforming is a thermodynamically attractive route for producing hydrogen that is not hampered by the sluggish kinetics of the OER.^5,18^ Although the amount of hydrogen that can be produced from glycerol is limited to ∼2 Mtons, the products of GOR have higher economic value than either glycerol, hydrogen, or oxygen. As a result, the profitability and sustainability of biodiesel production are improved, while simultaneously enhancing the efficiency of hydrogen production. Depending on photoanode material and reaction conditions, various high-value glycerol oxidation products, such as 1,3-dihydroxyacetone (DHA),^19,20^ glyceraldehyde,^21,22^ glyceric acid^23^ or formic acid,^24,25^ can be obtained.

**Scheme 1:**
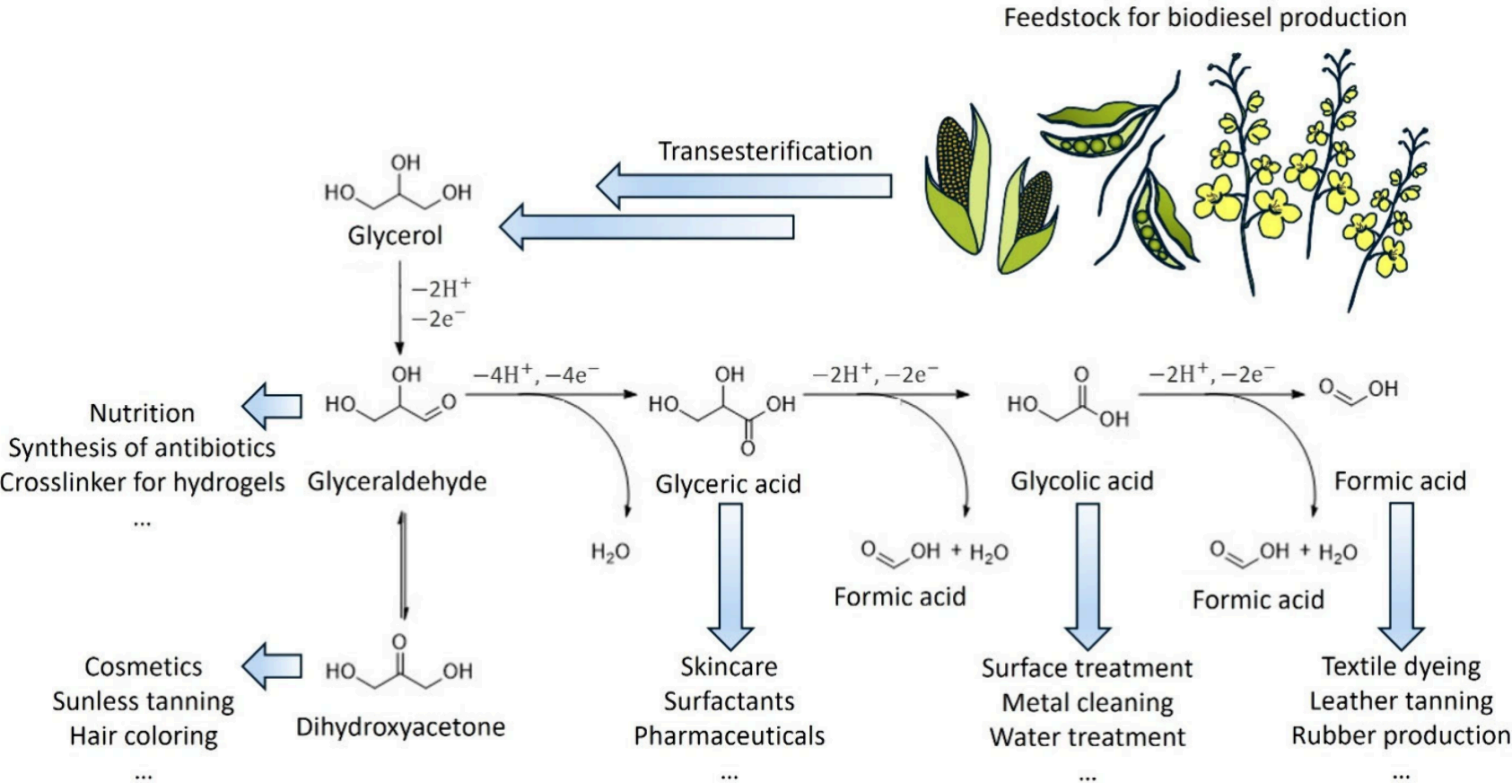
Possible Catalytic Pathways for the Glycerol Oxidation Reaction

Another, less studied, approach to improve the economic viability of PEC systems is the oxidation of 5-hydroxymethylfurfural (HMF). HMF is a biobased building block obtained from the dehydration of reducing sugars, and its oxidation products serve as sustainable precursors for bioplastics. Several studies have reported complete conversion of HMF to its fully oxidized form, 2,5-furandicarboxylic acid (FDCA), under dark electrolysis.^26^ However, achieving efficient oxidation under light illumination in PEC cells remains challenging, as it typically requires the use of mediators or molecular photocatalysts such as 2,2,6,6-tetramethylpiperidin-1-oxyl (TEMPO).^27^ The use of such mediators complicates product separation and, in the case of TEMPO, also limits light absorption due to its strong red-orange coloration. Therefore, further research is needed to optimize HMF oxidation within PEC systems.^28^

The construction of PEC cells generally includes several key components, each serving a specific function to ensure efficient reaction operation (see Figure 1). The central elements of a PEC cell are the electrodes, particularly the photoactive working electrode. This photoelectrode allows converting the solar energy into chemical energy and is typically made of n-type semiconductors such as TiO_2_, Fe_2_O_3_, BiVO_4_, or WO_3_ ^29^ or p-type semiconductors such as NiO, CuBi_2_O_4_, CuAlO_2_, or GaSe.^30,31^ The illumination of the photoanode leads to the excitation of electrons in the valence band (VB) to the conduction band (CB), thereby creating electron–hole pairs. These charge carriers are then separated and used for the redox reactions occurring at both electrodes of the PEC cell. The reduction of water molecules or protons to hydrogen gas (H_2_) takes place on the (photo)­cathode, while the oxidation reaction takes place on the (photo)­anode.^32^

**Figure 1:**
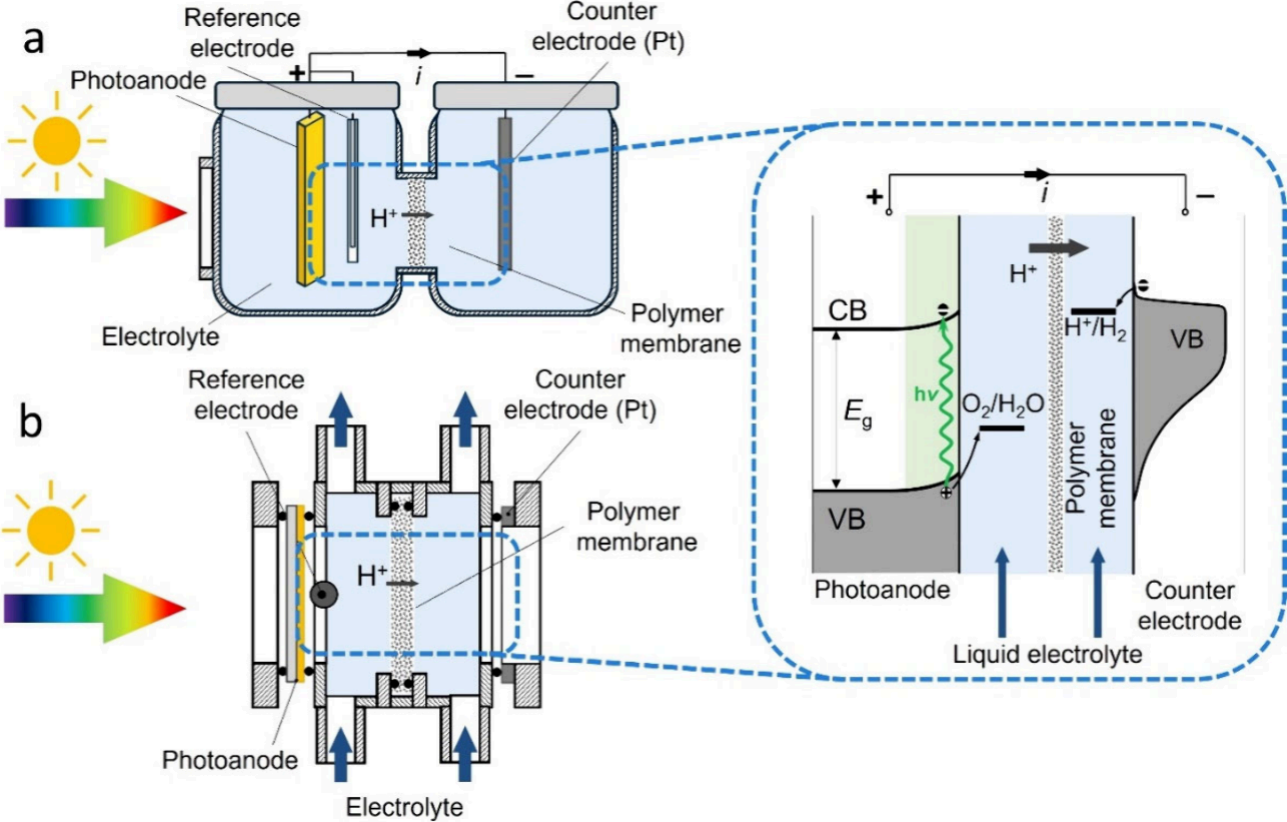
Schematic illustration of (a) an H-type PEC cell, (b) a flow PEC cell connected to a pump, and the water splitting process.

To prevent the mixing of hydrogen gas and the oxidation products, a membrane should be placed between the anodic and cathodic half cells. This separation also allows for the use of different pH conditions in the anodic and cathodic chambers, optimizing the performance of each photoabsorber/cocatalyst assembly. Hence, the membrane must possess excellent ionic conductivity properties to allow the protons generated at the photoanode during the oxidation reaction to pass through to the cathode, where they can be reduced to hydrogen gas. Proton exchange membranes (PEM), such as Nafion, are commonly used for this application.

Nafion is the first commercially produced perfluorosulfonic acid polymer, obtained by the copolymerization of tetrafluoroethylene and a perfluorovinylether, perfluoro-(4-methyl-3,6-dioxa-7-ostene-1-sulfonyl fluoride) (see Figure 2) and used as a proton conductor membrane in chlor-alkali-process, fuel, and PEC cells.^33^ These membranes were developed by DuPont (now Chemours) in the 1960s and have been continuously improved to meet specific market demands. Today, various types of Nafion membranes for electrolytic processes are available (Table 1), offering excellent mechanical properties, high chemical and thermal stability, and superior ion-conductive performance (Table 2).^34^

**Figure 2:**
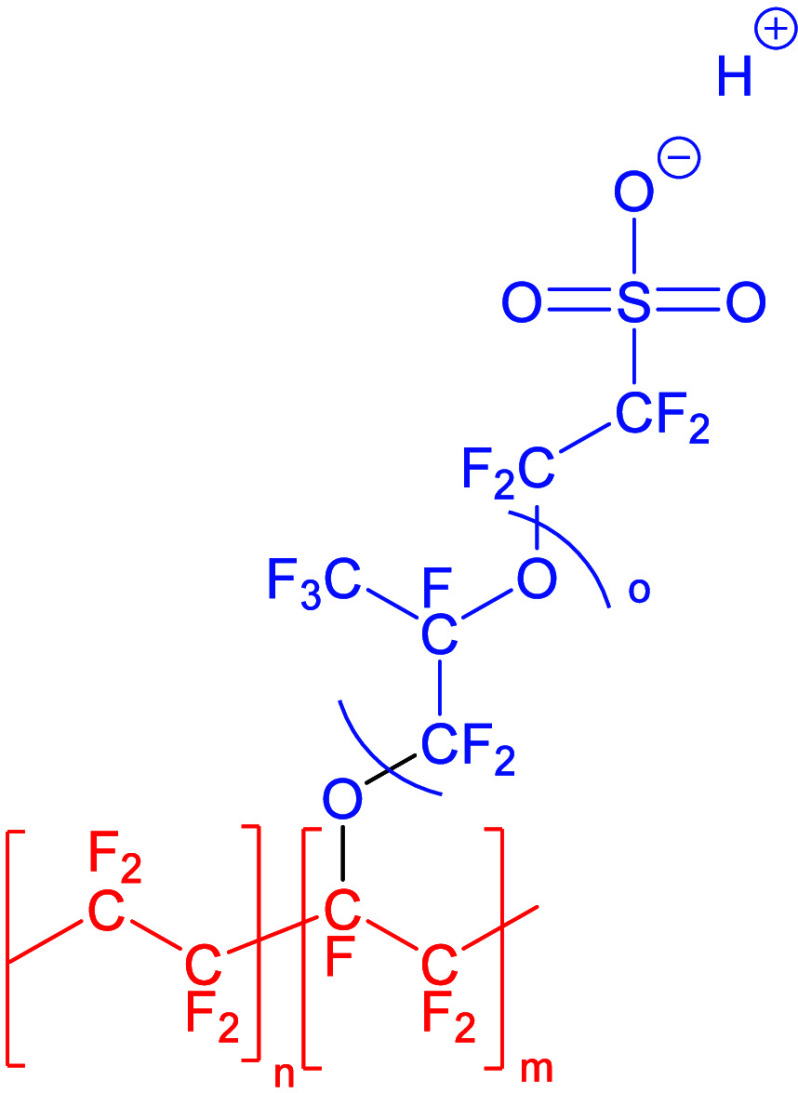
Schematic representation of the general structure of a Nafion proton exchange membrane depicting the hydrophobic perfluorinated backbone (red) and the more polar, hydrophilic perfluoroether side chain with the sulfonic acid end group (blue).

**Table 1:** Physical Parameters of Nafion Membranes^a^ ^,^ ^35^

membrane type	typical thickness (μm)	basis weight (g/m^2^)	density (g/m^3^)
Nafion N115	127	250	2.0
Nafion N117	183	360	1.98
Nafion N1110	254	500	–

a Measurements taken with the membrane conditioned to 23 °C, 50% RH.

**Table 2:** Physical Properties of Nafion 115/117/1110 Membranes^36–38^

property	typical value
proton conductivity (S/cm)	>0.100
water uptake (wt %)^b^	38
ion exchange capacity (mequiv/g)	>0.90
proton diffusion coefficient (cm^2^/s)	0.6 × 10^–6^
elastic modulus (MPa)^a^	249

a Measurements taken with the membrane conditioned to 23 °C, 50% RH.

b Water uptake from the dry membrane to the membrane after water soaking at 100 °C for 1 h.

The synthetic route for producing commercial membranes such as Nafion involves constructing a perfluorinated polymer backbone followed by the introduction of ionic side groups.^39^ The production of these perfluorinated polymers is both complex and costly, as it requires high-temperature polymerization under strictly controlled conditions, followed by extrusion or solution casting to obtain thin films.^40^ The manufacturing process frequently involves the use of organic solvents such as glycols or alcohols, which not only increase production costs but also contribute to global environmental pollution and significantly raise the ecological footprint of membrane fabrication.

For practical applications, a critical consideration is the high cost of photoelectrolyzers that operate with Nafion membranes, where the membrane itself represents a substantial portion of the total system cost.^41^ Currently, the market cost of Nafion-based membranes is about 1755 €/m^2^ (June 2025).^42^ A “dark” electrolyzer operated with renewable electricity is expected to have a capex cost of 1000 €/kW by 2030.^43^ For such an electrolyzer operating at 1 A/cm^2^, as reported in Figure 3, the estimated cost for equipping the device with Nafion membrane is around 100 €/kW,^33,34^ i.e. about 10% of the overall electrolyzer cost. While it is not trivial to determine the final cost of a photoelectrolyzer operating at much lower power density values, we can interpolate the data reported in Figure 3 and extrapolate the cost/power ratio for a Nafion membrane in a device operating instead at 10 mA/cm^2^, i.e. a typical working current density of a photoelectrolyzer. The χ^2^/(*N* – 1)-weighted interpolation shows that the power-normalized cost of the membrane drastically increases with decreasing current density, given the limited H_2_ production yield (thereby limiting the economic return of operating the photoelectrolyzer). At an operational current density of 10 mA/cm^2^, the costs for the membrane would be about 1890 €/kW. Moreover, the performance of Nafion membranes is negatively affected by operations under harsh conditions. Nafion is not appropriate for processes running below 0 °C or above 100 °C, and its conductivity properties strongly depend on relative humidity (RH), i.e. presence of water in hydrophilic domains. Furthermore, Nafion is stable in hydrogen peroxide (H_2_O_2_) solutions up to 30%, while its decomposition products, OH and OOH radicals, damage the sulfonate group (−SO_3_^–^) and lead to the degradation of the membrane.^30^ Another limitation of Nafion is it is high-cost and the challenges associated with its recycling, primarily due to its nonbiodegradability. This characteristic raises significant environmental concerns and contributes to its overall ecological footprint.

**Figure 3:**
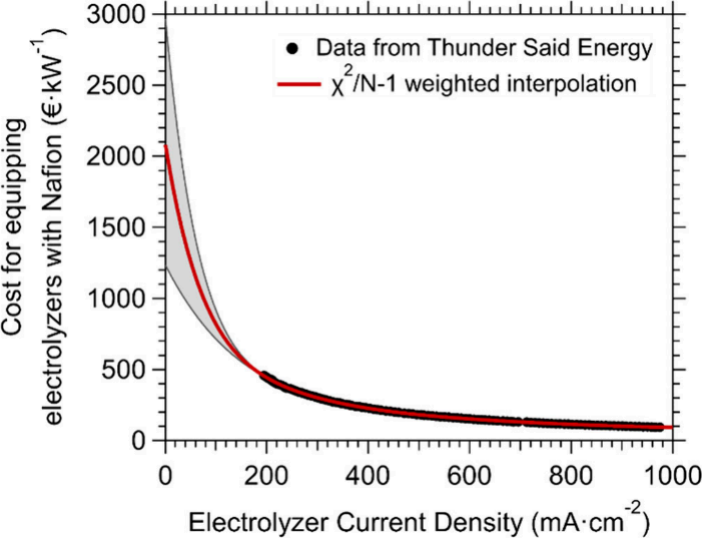
Power-normalized cost of equipping an electrolyzer with Nafion membranes as a function of the electrolyzer current density. The data are taken from a model developed by Thunder Said Energy, taking the cost of Nafion at 2000 $/m^2^ (∼1775 €/m^2^).^42^ The data have been linearly interpolated to extrapolate the membrane cost at the current densities relevant for PEC hydrogen production (using nonconcentrated sunlight). The shaded area identifies a series of polynomial interpolations with χ^2^/(*N* – 1) values ranging between 1.36 and 0.99. The red curve has been obtained by weighted-averaging the different interpolations using the χ^2^/(*N* – 1) values as weights.

Technological innovations, such as PEC devices, can only truly be sustainable if they are based on sustainable materials, that do not pose negative effects on the environment or on human health. According to the EU Commission in 2024, the use of per- and polyfluoroalkyl substances (PFAS), such as Nafion and other perfluorinated ion exchange membranes (IEMs), should be partly or completely restricted.^44,45^ As a consequence, the electrolyzer industry must also adapt by developing and adopting new membrane technologies that are both effective and environmentally benign, ensuring compliance with future regulations and contributing to a healthier planet. This draws attention to the importance of focusing on the development and invention of new IEMs for (photo)­electrochemical applications retaining similar physical, chemical, and thermal properties, as Nafion, however with improved recyclability and degradation characteristics.

IEMs used in PEC cells have distinct property requirements compared to membranes employed in dark electrolyzers. PEC cells typically operate at lower current densities, generally not exceeding 30 mA·cm^–2^, whereas dark electrolyzers often operate at current densities at or above 1 A·cm^–2^. This difference has several implications for membrane performance. Lower current densities in PEC cells result in reduced ohmic losses across the membrane and moderate ionic conductivity is generally sufficient, in contrast to dark electrolyzers, where high ionic conductivity is essential to minimize voltage losses. Additionally, the lower currents reduce electrochemical stress on the membrane, thereby decreasing degradation associated with ion flux and applied potential. Thermal management is also less demanding in PEC systems due to reduced Joule heating. However, PEC membranes are exposed to a combination of long-term illumination and relatively harsh reaction environments, which can induce photodegradation of the polymer material, potentially decreasing its mechanical, chemical, and ionic transport properties, and ultimately affecting device performance. Consequently, polymer materials for PEC IEMs should exhibit high photochemical stability, resist localized oxidative stress from photogenerated radicals, and maintain robust operational properties under ambient temperature and pressure. Furthermore, optical transparency is a critical requirement to allow effective illumination of the photoelectrodes.

This review examines potential alternative materials for polymeric membranes, focusing on their synthesis, modification, and characterization. It highlights the most promising candidates that could replace PFAS-based membranes in PEC cells, offering reduced production and operational costs while providing sustainable solutions to mitigate environmental pollution. The membranes are classified according to their polymer backbones, and their properties are systematically discussed, incorporating recent advances in the field. A detailed overview of characterization techniques is provided, including electrochemical impedance spectroscopy and hard X-ray spectroscopy under ambient pressure conditions. Additionally, the review explores the integration of artificial intelligence (AI) and machine learning (ML) approaches in membrane research, emphasizing their potential to accelerate material discovery and optimization. Finally, the influence of membrane properties on overall device performance is analyzed, highlighting the critical role of continued research in developing high-performance, sustainable membrane materials for PEC applications.

## 2 Ion Exchange Membranes in PEC cells

The following chapter focuses on the definition and classification of ion exchange membranes (IEMs), with particular emphasis on their polymer backbones. It provides an overview of the most promising polymer categories reported in the literature as suitable candidates for (photo)­electrochemical applications, including aromatic polymers and biopolymer-based systems. Unfortunately, due to the large number of potential polymer candidates, it is not possible to include an exhaustive list or provide a detailed overview of all materials within the scope of this review. In addition, the chapter discusses various polymer modification strategies, such as sulfonation, grafting, cross-linking, blending, and incorporation of inorganic fillers, highlighting their impact on key membrane properties, including ionic conductivity, mechanical and thermal stability, and selectivity.

IEMs are essential in energy conversion and storage applications, including diffusion dialysis, electrodialysis, redox flow batteries, fuel cells, and (photo)­electrochemical cells. They are specialized semipermeable membranes that make the transport of specific ions between separated cells possible while blocking others. IEMs typically consist of a hydrophobic backbone matrix with an attached side chain containing a functional ionic group. Depending on the type of functional group, they can be classified into cation exchange membranes (CEMs), anion exchange membranes (AEMs), and proton exchange membranes (PEM). CEMs allow the passage of positively charged monovalent cations, e.g., Na^+^, and K^+^, and contain negatively charged functional groups, such as −SO_3_^–^, −COO^–^, −PO_3_^2–^, −PO_3_H^–^, and −C_6_H_4_O^–^. These anionic sites create electrostatic repulsion toward multivalent cations, *e*.*g*., Ca^2+^, and Mg^2+^, due to their higher charge density and larger hydration shells. This phenomenon, known as dielectric exclusion, together with size exclusion and the Donnan effect—an electrostatic interaction where ions with charges opposite to those of the membrane are attracted and accumulate near the membrane surface, while like-charged ions are repelled—contributes to the membrane’s selective permeability and suppression of undesired ion transport.^46,47^ A specific type of CEM is the PEM, which selectively transports protons, *i*.*e*., H^+^ ions and is widely used in fuels cells.^48^ Analogously, AEMs contain positively charged functional groups such as −NH_3_^+^, −NRH_2_^+^, −NR_2_H^+^, −NR_3_^+^, and −PR_3_^+^ and allow the passage of monovalent anions but reject cations and multivalent anions.^49,50^ At the same time, the degree of functionality, defined as the number of functional groups per repeating unit in the polymer chain, can significantly influence membrane properties such as ion exchange capacity (IEC), ionic conductivity, water uptake (WU), and stability. An increase in functionality leads to a higher IEC, as more functional groups become available for ion transport.^51^ Furthermore, an excessively high degree of functionality can lead to membrane degradation due to ionic interactions with other molecules, resulting in dissolution in water and the hydrolysis of functional groups over time, which compromises membrane durability. Therefore, finding the appropriate balance in functionality is essential to ensure optimal membrane properties.

In addition to chemical modification, surface modification methods can also have a significant impact on the membrane properties. Various techniques, including plasma treatment, UV irradiation, coating and electrodeposition, can greatly enhance ion conductivity, permselectivity, and hydrophilicity.^52–54^ However, a major research challenge remains in developing more effective modification strategies and identifying the most efficient materials for optimization.^55^

To be applied in a wide range of applications, IEMs for PEC cells are required to possess excellent characteristics, including high ionic conductivity, selective ion permeability, strong mechanical and thermal stability, low electrical resistance, good chemical stability under both acidic and alkaline conditions, as well as viable production cost. Typical reaction conditions for water splitting in a PEC cell encompass a temperature range between 20 and 80 °C, a cell voltage between 1.8 and 2.2 V, a light intensity of 1000 W/m^2^ (at AM1.5G illumination conditions), and a pH value ranging from 1 to 14 depending on the utilized electrolyte. Figure 4 illustrates the classification of IEMs based on their constituent materials, specifically distinguishing between perfluorinated and nonfluorinated polymers. The nonfluorinated materials include bio- and bioinspired polymers as well as hydrocarbon-, aromatic-, and composite-based blends.

**Figure 4:**
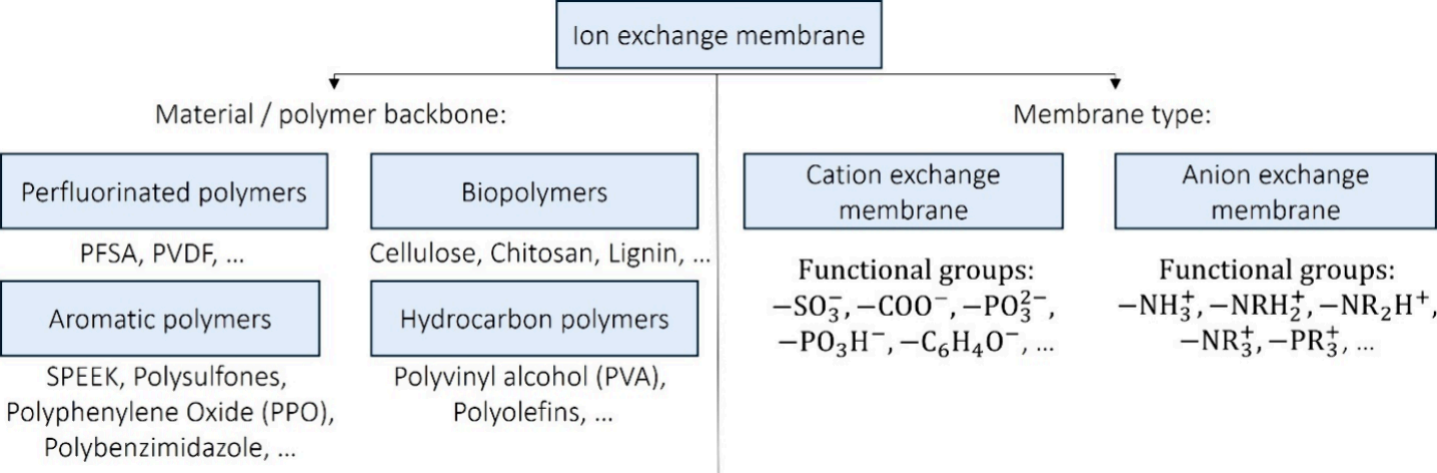
Classification of membranes based on materials/polymer backbone which is modified.

Recently, there has been growing interest in ion exchange membranes (IEMs) made from sustainable materials or those produced and functionalized using environmentally friendly methods, offering excellent properties for (photo)­electrochemical applications.

Numerous studies have reported the use of sulfonated aromatic polymers as potential alternatives to fluorinated membranes, owing to their favorable ion exchange properties and cost-effectiveness. Significant research efforts have focused on aromatic materials such as sulfonated polyimide (SPI), sulfonated polyether sulfone (SPES), sulfonated polyphenylene oxide (SPPO), and sulfonated polyether ether ketone (SPEEK), all of which exhibit promising proton conductivity and chemical stability for electrochemical applications.^56^ SPEEK-based membranes have attracted significant research interest due to their excellent thermal stability and mechanical properties.^57–59^ The proton conductivity of these membranes can be precisely controlled by adjusting the degree of sulfonation. However, a major drawback is that highly sulfonated SPEEK membranes exhibit excessive swelling at elevated temperatures (>80 °C), which can lead to structural failure and compromised performance.^60^ To address this challenge, Zhang et al.^61^ demonstrated the fabrication of ultrathin, highly ordered SPEEK proton exchange membranes using a Langmuir–Blodgett self-assembly process. This approach results in membranes with enhanced ion conductivity, while the ultrathin structure effectively limited water uptake and swelling, thereby improving the dimensional stability of the membrane.

In this review, we focus on aromatic polymer-based membranes and composite membranes derived from aromatic polymers, particularly polysulfone and polyphenylene oxide, as well as biopolymer-based membranes. These materials are among the most promising candidates for use in (photo)­electrochemical cells due to their structural stability and excellent mechanical and thermal properties.^62–63,64^ At present, it is not possible to provide precise estimates of membrane production costs or final prices, as the studies discussed herein are conducted at the technology development stage, involving experimental optimization, material synthesis, and performance testing. Accordingly, the current technology readiness level (TRL) of these membranes is approximately 3–4, based on the NASA evaluation framework.^65^

### 2.1 Aromatic Polymers

#### 2.1.1 Polysulfones

Polysulfones (PSU) are excellent material candidates for use as ion exchange membranes in PEC devices due to their good mechanical strength, excellent film-forming properties, and outstanding oxidative, thermal, and hydrolytic stability.^66,67^ Several studies have reported the functionalization of polysulfones by introducing an anionic sulfonic group through simple sulfonation or under UV irradiation, which facilitates subsequent modification with other groups.^68,69^ The sulfonated polysulfone (SPSU) membranes with different degrees of sulfonation (11.6%, 69.1%, 94.1%, and 105.9%) exhibited higher IEC compared to the commercial Nafion membranes, as well as an increase in WU with the increasing degree of sulfonation and higher tensile strength up to 55.8 MPa.^68^ Despite these promising properties, the proton conductivity of SPSU membranes limited to about 5.0 × 10^–2^ S/cm, which is significantly lower than that of Nafion due to their higher molecular rigidity of SPSU. This rigidity, however, leads to higher tensile modulus (Young’s modulus) in both dry and wet conditions. In 2009, Wang et al. reported the synthesis and characterization of polysulfone-based alkaline AEMs with high ionic conductivity by introducing a chloromethyl pendant group into polysulfone, creating chloromethylated polysulfone (CMPSU), followed by quaternization, a chemical process in which amine groups are converted into quaternary ammonium groups to enable anion exchange, and alkalization.^70^ Their study demonstrated that reaction time and temperature significantly influenced the chloromethylation, i.e. functionalization, processing and gelation. The developed alkaline AEM exhibited an ionic conductivity of up to 3.1 × 10^–2^ S/cm at room temperature, which increased to 7.33 × 10^–2^ S/cm with rising temperature. Furthermore, the AEM remained stable in concentrated base solution of up to 8.0 M KOH at room temperature. The quaternization process was studied with various aliphatic diamine compounds, and it was demonstrated that membrane properties could be optimized by adjusting the type and amount of diamine in the polymer casting solution. The effect of diamine chain length revealed that quaternization with diamines having longer aliphatic chains (alkyl groups bonded to the amine nitrogen) required a lower excess of diamine to produce membranes with low electrical resistance and high permselectivity. Komkova et al.^71^ compared amines with different aliphatic chain lengths, including *N,N,N′,N′*-tetramethylhexanediammonium (TMHDA), *N*,*N*,*N*′,*N*′-tetramethylethylenediammonium (TMEDA), *N*,*N*,*N*′,*N*′-tetramethylbutanediammonium (TMBDA), *N*,*N*,*N*′,*N*′-tetramethylmethanediamine (TMMDA), and *N*,*N*,*N*′,*N*′-tetramethyl-1,3-propanediamine (TMPDA). Their study demonstrated that AEMs containing diamines with shorter aliphatic chains are less stable and, thus, are prone to faster degradation. Generally, the cross-linking reaction enhances mechanical and thermal stability of the polymer, while also improving conductivity and selectivity.^72,73^ Scheme 2 reports a diagram of the possible chemical modifications of polysulfones described above.

**Scheme 2:**
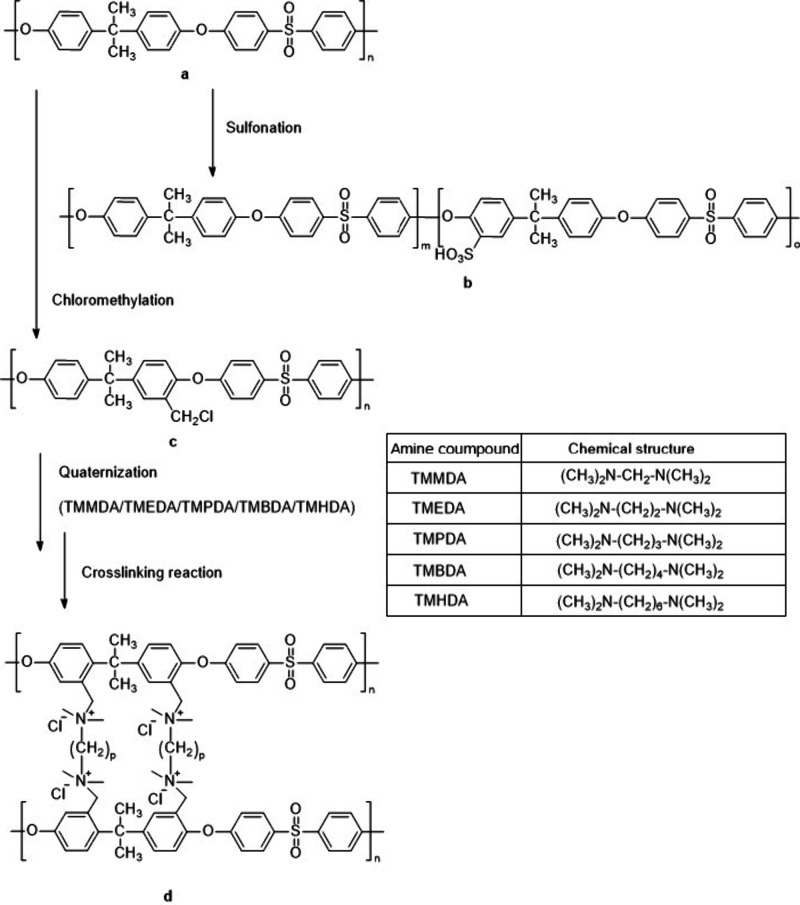
Schematic Diagram Illustrating Possible Modifications of Polysulfones: Sulfonation of PSU (a) to SPSU (b) and Chloromethylation of PSU (a) to CMPSU (c) Followed by a Quaternization Reaction Using Diamines with Aliphatic Chain Groups of Varying Lengths and a Cross-Linking Reaction to Produce AEM (d)

Additionally, many studies have reported the mixing of ion exchange membranes (IEMs) with inorganic fillers such as TiO_2_, Al_2_O_3_, or SiO_2_ to enhance thermal stability and ionic conductivity.^74–77^ Nonjola et al.^78^ investigated the effect of incorporating TiO_2_ nanoparticles into quaternary polysulfone (QPSU) to create a nanocomposite membrane. Their findings demonstrated that the addition of an inorganic filler to the polysulfone-based anion exchange membrane (AEM) improved thermal resistance and increased the WU. Furthermore, Vinodh et al.^79^ showed that using a quaternized polysulfone membrane with zirconia (ZrO_2_) filler led to enhanced electrochemical performance, with a maximum power density of 250 mW/cm^2^ observed for QPSU/10% ZrO_2_ at 60 °C.

Choi et al.^80^ reported the fabrication of a nanofiber network-based membrane using sulfonated poly­(arylene ether sulfone), demonstrating that manipulating polymer morphology at the nanoscale can significantly enhance membrane functionality and mechanical properties. The membrane was fabricated through a three-dimensional interconnected network of electrospun polyelectrolyte nanofibers, with the interfiber voids filled with an inert, uncharged polymer. This approach enables the independent selection of the polyelectrolyte and inert polymer, allowing for the precise control of fiber diameter and fiber volume fraction, which directly influences the membrane’s physicochemical properties. By optimizing these parameters, the membrane exhibits improved mechanical strength while effectively preventing excessive swelling, making it a promising strategy for the development of high-performance ion exchange membranes.

A promising research direction involves the development of biosourced polysulfones as sustainable alternatives to conventional polysulfones synthesized from petroleum-derived monomers. The utilization of renewable, biobased monomers offers the potential to significantly reduce the environmental footprint while preserving the structural integrity required for subsequent functionalization and application as IEMs. In this context, several studies have identified lignin-derived aromatic compounds as valuable precursors for the synthesis of biosourced polysulfones.^81,82^

#### 2.1.2 Poly­(phenylene oxide)

Poly­(2,6-dimethyl-1,4-phenylene oxide) (PPO) represents a polyaryl with excellent electrical properties, high resistance to acids and bases as well as other chemicals, excellent dimensional stability, low moisture absorption, high mechanical and dielectric strength, and high glass transition temperature.^83^ PPO can be functionalized in various ways, such as sulfonation and bromination, for use as an IEM. Sulfonation can be performed using chlorosulfonic acid (HSO_3_Cl) as the sulfonating agent, with the sulfonic groups attaching directly to the phenylene ring resulting in sulfonated PPO (SPPO), while the sulfonation rate is directly proportional to the concentration of the phenylene repeating unit and concentration of chlorosulfonic acid.^84^ The bromination of PPO can occur either directly on the benzene rings or on one of the methyl groups attached to the benzene ring, resulting in bromomethylated PPO (BrPPO). Rong-Qiang Fu et al.^83^ described the preparation of PPO-based acid–based polymer blend membranes by mixing solutions of SPPO/*N*-methyl-2-pyrrolidone (NMP)/*n*-propylamine (PrNH_2_) and BrPPO/NMP/PrNH_2_, followed by casting on glass Petri dishes and acidifying with hydrochloric acid. The resulting polymer blend exhibited ionic cross-linking between the sulfonic groups of SPPO and the amine groups of aminated BrPPO (as illustrated in Scheme 3), which was confirmed by comparing experimental and theoretical IEC values. The proton conductivity of the blend membranes was found to be similar to that of Nafion 117, while ionic cross-linking led to a more compact membrane structure, that reduced water absorption and swelling even at higher temperatures. The membrane with 30% BrPPO revealed the lowest proton conductivity of 6.9 × 10^–2^ S/cm, whereas the proton conductivity increased with a higher proportion of SPPO, reaching a peak value of 9.4 × 10^–2^ S/cm for pure SPPO membranes at 25 °C. Furthermore, all membranes exhibited lower methanol crossover compared to Nafion 117, with a decrease in methanol crossover as the BrPPO content increases, while also demonstrating good mechanical properties and oxidative stability.

**Scheme 3:**
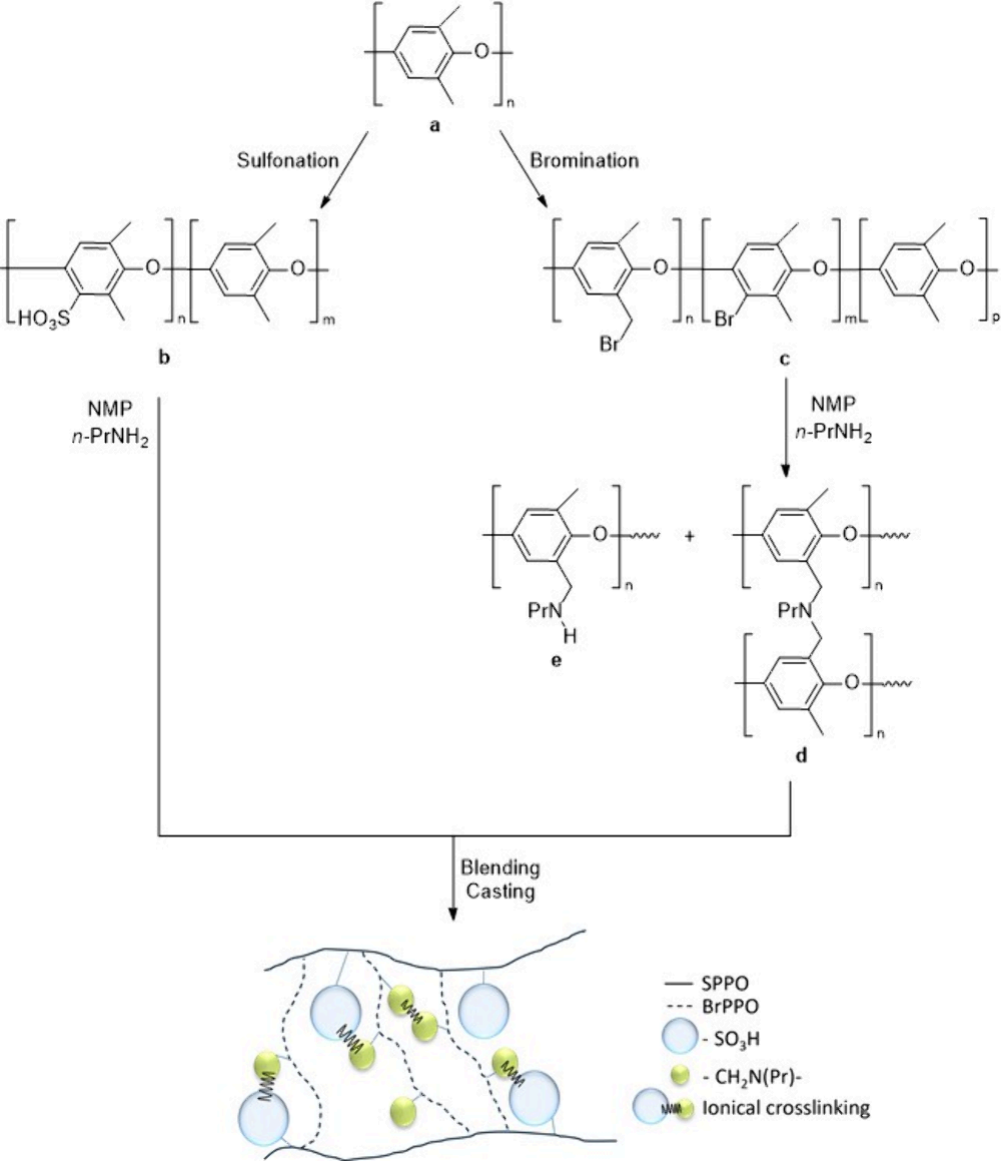
Schematic Representation of the Preparation of the Acid–Base Polymer Blend Membranes: Sulfonation of PPO (a) to SPPO (b) and Bromination to BrPPO (c) Followed by Blending and Casting^a^

a Adapted with permission from ref 83. Copyright 2008 Elsevier.

In 2021, Tsehaye et al.^85^ reported a synthetic method for incorporating *N*-spirocyclic quaternary ammonium (QA) cations into a PPO membrane backbone using a rapid UV-irradiation approach. The process involved a two-step functionalization of PPO with methylbenzyldiallylammonium groups (PPO-Q), followed by cross-linking with *N*,*N*-diallylpiperidinium chloride (DAPCl) through UV-induced free-radical polymerization substitution reactions, while a photoinitiator Irgacure 2959 was used for casting and cross-linking reaction (Figure 5). A total of five membranes, with different IEC values ranging from 1.5 to 2.8 mmol of Cl^–^ g^–1^, achieved by adjusting the ratio of PPO-Q to DAPCl, were produced and characterized. Tsehaye et al. found that membranes with higher IEC values exhibited increased water uptake, enhanced chloride mobility, and reduced membrane resistance. However, this high-water uptake negatively impacted the cyclability due to active species crossover during the cycling test in aqueous organic redox flow battery (AORFB) using TMA-TEMPO/methylviologen as the active materials.

**Figure 5:**
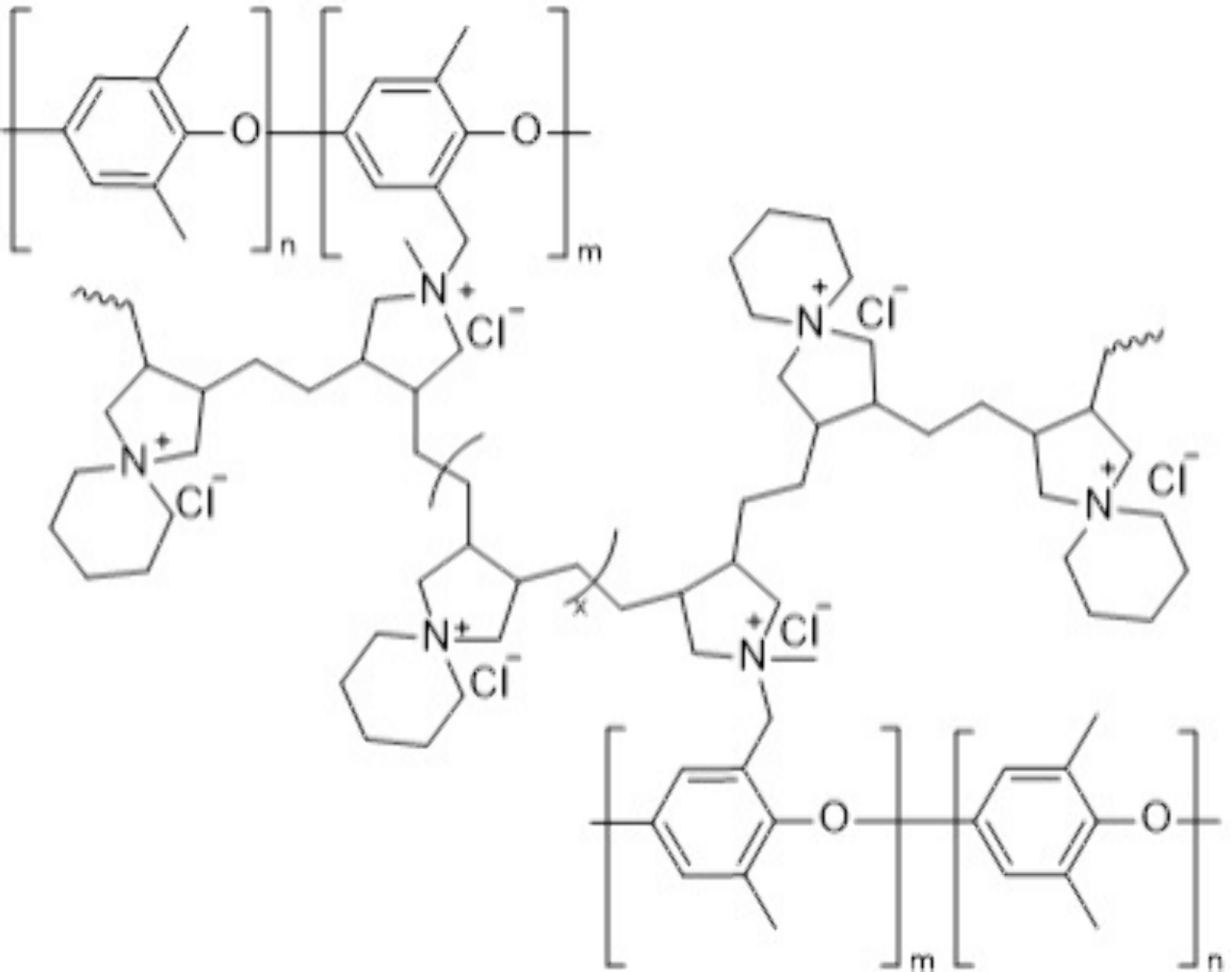
Schematic representation of a PPO-based cross-linked AEM. Adapted from ref 85. CC BY 4.0.

### 2.2 Biopolymers

Biopolymer-based IEMs are becoming an interesting and promising sustainable alternative to synthetic-based IEMs due to their availability, environmental compatibility, adaptability for modification, and low costs. Cellulose is the most abundant organic polysaccharide consisting of d-anhydroglucose (C_6_H_11_O_5_) repeating units joined by β-(1,4)-glycosidic linkages at carbon C-1 and C-4 carbon positions, with a total production capacity of 10^11^–10^12^ t each year.^86,87^ It can be derived from plant biomass, algae, and cellulose-producing bacteria and offers excellent mechanical strength, good water retention capacity, low permeability to gases and methanol, and good thermal stability. Another very common biopolymer is chitin, which can be extracted from crustacean animals and converted into chitosan through deacetylation.^88^ Chitosan is a linear polysaccharide consisting of randomly distributed β-(1–4)-linked d-glucosamine and *N*-acetyl-d-glucosamine and has high water affinity because of the presence of three different polar functional groups: −OH, −NH_2_, and −C–O–C–.^88^ Moreover, lignin has been reported in several studies as a good candidate for use as a basis for IEMs.^89–91^ It is conceivable to modify these biopolymers through functionalization to increase the ion conductivity properties and to enable their use as IEMs. Additionally, incorporating of the mentioned biopolymers into blend membranes by combining different polymers can provide IEMs with advantageous properties.

In 2009, Seo et al.^92^ successfully prepared and characterized cellulose-based PEMs using sulfosuccinic acid (SA) as a cross-linking agent (5 to 30 wt %, Scheme 4). The authors demonstrated that both proton conductivity and IEC increased in proportion to the SA content in the membrane. The thermal stability also increased for membranes containing up to 30 wt % SA. In addition to excellent thermal stability up to 250 °C, the cellulose/SA membranes exhibited enhanced mechanical properties, i.e. maximal tensile strength with a reported break pressure of 5.649 MPa. Due to the largest content of acidic −SO_3_^–^ groups, the membrane with 30 wt % content of SA showed the highest room-temperature value of the proton conductivity of 0.023 S/cm, compared to a conductivity of 10^–3^–10^–2^ S/cm for membranes with lower SA content.

**Scheme 4:**
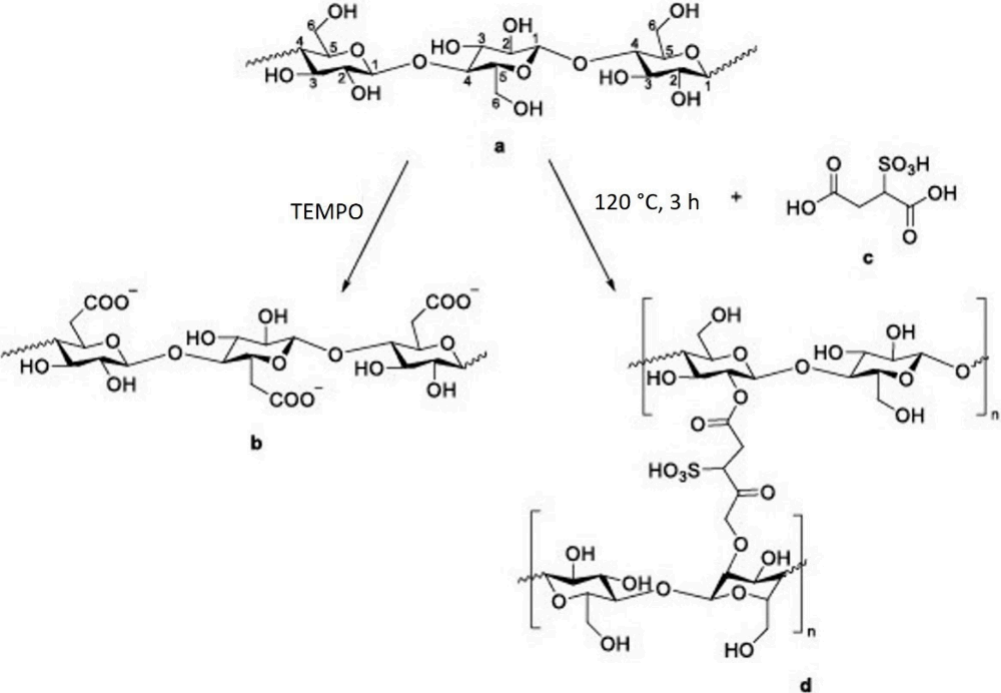
Schematic Diagram Illustrating Possible Modifications of Cellulose: Oxidation Reaction of Cellulose (a) to Carboxylated Cellulose (b); Preparation of cellulose-based PEM (d) through Cross-Linking Reaction of Cellulose (a) with Sulfoccinic Acid (c)^a^

a Adapted with permission from ref 92. Copyright 2009 Springer.

Several studies have reported the use of cellulose nanocrystals (CNCs) in the preparation of highly ion-selective membranes for applications in redox flow batteries (RFBs) and fuel cells (FCs).^93,94^ The synthesis of cellulose nanocrystals *via* acid hydrolysis with sulfuric acid partially converts hydroxyl (−OH) groups to sulfate ester (−OSO_3_^–^) groups, thereby enhancing the proton conduction properties of the membranes.^95^ Furthermore, the incorporation of sulfonate ester modified cellulose nanocrystals into membrane matrices enhances their mechanical stability and durability, ensuring long-term performance under operational conditions. For application at high temperatures and dry conditions, the CNCs can be doped with heterocyclic molecules, particularly imidazole, which leads to enhanced proton conductivity up to 0.027 × 10^–2^ S/cm at 140 °C, which is about 5 orders of magnitude higher than that of pure CNC, but 2 orders of magnitude lower than that of cellulose/SA composite membrane.^96,97^

The substitution of C-6 hydroxyl groups with anionic carboxylate (−COO^–^) groups in cellulose can be achieved through an oxidation reaction under alkaline conditions, using a TEMPO radical catalyst, sodium bromide (NaBr), and sodium hypochlorite (NaClO) as the oxidant.^98–100^ The resulting carboxylated cellulose membrane exhibits significantly lower (10 to 50×) proton conductivity compared to commercial Nafion membranes but demonstrates enhanced gas barrier properties and reduced sensitivity to changes in relative humidity (RH), due to its ability to bind water molecules strongly, allowing the membrane to retain conductivity even at lower RH levels.^98^

In addition to the hydroxyl group, chitosan contains an amino (−NH_2_) group that can be modified to enhance its electrochemical performance. Without modification or cross-linking, chitosan exhibits a conductivity of approximately ∼10^–9^ S/cm at room temperature, mainly due to limitations in hydrogen ion movement.^101^ However, modification methods like sulfonation, phosphorylation, quaternization, or chemical cross-linking are generally less effective when compared to more complex methods used for preparing composite membranes. In composite membranes, the inorganic component provides excellent mechanical and thermal stability, while the organic material contributes enhanced chemical reactivity, flexibility, and processability. Jie Wang et al.^101^ reported for the first time in 2018 the use of carbon nanotubes (CNTs) as an additive to modify the chitosan-based proton exchange membrane and showed improved proton conductivity of 0.044 S/cm at 80 °C.

To summarize, the key findings from this overview of earlier research show that there are potentially many alternatives to Nafion for IEMs that can be applied in (photo)­electrochemical applications, as shown in Table 3. The most critical requirements for new IEMs can be summarized as follows:

- Moderate ionic conductivity, sufficient for typical PEC operating current densities (<30 mA·cm^–2^)
- High chemical, mechanical, and thermal stability to ensure reliable operation under aqueous electrochemical conditions
- Long-term durability during repeated operation
- Photochemical stability under continuous illumination, including resistance to photodegradation by UV light and photogenerated radicals
- Optical transparency, enabling efficient illumination of photoelectrodes in certain PEC cell configurations
- Cost-effective and sustainable production, minimizing environmental impact.

**Table 3:** Properties of Selected Examples of Alternative Materials for IEMs^d^

polymer	modification/type	σ (S/cm)	power density (mW cm^–2^)	IEC (mmol g^–1^)	WU (%)	thermal stability (°C)	ref(s)
Nafion 117	PFSA	0.081	97	>0.9	38	280	^36–38^
PSU	DS, 69.1%	0.004	–	1.39	34.1	–	^68^
	DS, 94.1%	0.034	–	1.82	42.4	–	^68^
	DS, 105.9%	0.044	–	2.01	48.9	–	^68^
	DS, 111.6%	0.048	–	2.10	49.5	–	^68^
	chloromethylation	0.073	–	–	–	–	^70^
	Q/TMMDA	–	–	0.15^a^	–	–	^71^
	Q/TMEDA	–	–	1.46^a^	–	–	^71^
	Q/TMPDA	–	–	1.30^a^	–	–	^71^
	Q/TMBDA	–	–	0.87^a^	–	–	^71^
	Q/TMHDA	–	–	1.33^a^	–	–	^71^
	C/ClNH_2_	0.100	–	1.5^a^	40	222	^73^
	C/HEXCl	0.100	–	1.7^a^	50	219	^73^
	QPsf/TiO_2_ 2.5%	0.085	–	–	32	190	^78^
	QPsf/TiO_2_ 10%	0.125	–	–	39	190	^78^
	QPSU/ZrO_2_ 10%	0.015	250	0.92^a^	19	≥150	^79^
PPO	SPPO-0	0.094	–	–	64	230	^83^
	BrPPO-30	0.069	–	–	29	230	^83^
	UV-FRP (M1.7)	0.002^b^	–	1.71^a^	37	≥200	^85^
biopolymer	cellulose CBM-30	0.023	–	0.53^a^	<20	≥250	^92^
	NCC-PVA-T5	0.013^c^	–	–	–	244	^93^
	CNF–COOH	≥0.001	–	–	>50	–	^98^
	CS/CNT	<0.02	48	–	≥75	200	^101^

a mequiv g^–1^.

b Cl^–^ ion conductivity.

c At 120 °C.

d PSU, Polysulfones; DS, degree of sulfonation; Q, quartenization; C/ClNH_2_, cross-linking of the chlorosulfonated polyarylene ether sulfone (PES) with NH_2_–PES; C/HEXCl, cross-linking of the PES with hexane diamine; QPsf/TiO_2_,quaternary polysulfone/Titanium dioxide nanocomposite membrane; QPSU/ZrO_2_, zirconia incorporated quaternized polysulfone membrane; PPO, polyphenylene oxide; SPPO-0, sulfonated PPO-based acid–base polymer blend membranes; BrPPO-30, bromomethylated PPO-based acid–base polymer blend membranes with 30 wt % of BrPPO; UV-FRP (M1.7), cross-linked membranes based on PPO synthesized via UV-induced free radical polymerization; Cellulose CBM, cross-linked polymer blend membranes based on cellulose and sulfosuccinic acid (30 wt %); NCC-PVA-T5, nanocrystalline cellulose poly­(vinyl alcohol)-triazole5 composite membrane; CNF–COOH, carboxylated cellulose nanofiber-based membranes; CS/CNT, membranes based on chitosan and solvent-free carbon nanotube fluids.

Unlike dark electrolyzers, PEC cells generally operate at ambient temperature and pressure. Nevertheless, most IEMs require activation prior to use to ensure optimal ionic conductivity, which is commonly achieved under harsh conditions such as boiling in acid. This step is necessary to fully protonate or hydrate the ionic groups within the membrane, which makes thermal and chemical stability during pretreatment an important requirement. Importantly, the photocurrent density in PEC cells is typically up to 30 times lower than in dark electrolyzers. As a result, membranes in PEC cells are subjected to much lower ion fluxes and ohmic losses, which means that moderate ionic conductivity is sufficient and extremely high conductivity, as required in dark electrolyzers, is not essential. However, the membranes must still maintain chemical stability to resist degradation by reactive species generated in the electrolyte and at the electrodes, and mechanical robustness to withstand swelling, handling, and cell assembly stresses.

The most promising candidates include aromatic polymers, such as polysulfones and polyphenylene oxide, as well as IEMs based on biopolymers like cellulose or chitosan. The properties of these polymers can be tailored through surface modifications, the substitution of functional groups, or cross-linking with other polymers. It is also essential to use sustainable methods for membrane fabrication and to ensure environmentally friendly approaches. Numerous studies over the past decade have investigated various synthesis methods and explored the impact of different factors on membrane properties, revealing a broad area for further investigation.

## 3 Sustainable Approaches for Membrane Preparation and Functionalization

The conventional fabrication of IEMs often involves aggressive chemical treatments, such as sulfonation with fuming sulfuric acid (H_2_SO_4_) or chlorosulfonic acid (HSO_3_Cl). Although these methods are efficient, they produce hazardous waste and present significant environmental and safety risks. The fundamental principles of green chemistry were established by Paul Anastas and John Warner in 1998 emphasizing waste prevention, atom economy, safer solvent selection, energy efficiency, renewable feedstocks, and degradation of end products. Additionally, they promote designing safer chemicals, reducing derivative use, real-time analysis for pollution prevention, and inherently safer processes.^102^ To promote greener chemistry, researchers are exploring alternative synthesis routes that reduce toxic byproducts while maintaining membrane performance. This chapter highlights sustainable and innovative approaches to IEM fabrication, focusing on eco-friendly methods that balance functionality with environmental responsibility and summarizes alternatives pathways for IEM functionalization in Table 4.

**Table 4:** Selected Examples of Sustainable Sulfonation and Bromination Pathways for IEM Functionalization^a^

functionalization agent	reaction conditions	solvent	mechanism	yield (%)	ref(s)
Sulfonation					
SO_3_	HHP	SF	S_E_Ar	–	^105^,^106^
K_2_S_2_O_5_	ambient *T*, *p*	H_2_O, HFIP	HAS	86	^107^,^108^
HSO_3_^–^	–	–	S_N_Ar	–	^103^
SO_2_, CH_4_N_2_O_2_S	Pd catalyst, 100 °C	DMSO	[M]	87	^109^
Bromination					
KNO_3_, HBR	NP, 25 °C	SF	*in situ*	91	^110^
NaBr, KNO_3_, HCl	NP, 25 °C	SF	*in situ*	–	^110^
NBS	35 °C	ACN/H_2_O	LA/LB	78	^112^
NBS	55 °C, *h*ν	ACN	RH	77	^116^
TBCA	reflux/6 h	EtOAc	BB	84	^118^
TBBDA	reflux/1.5 h	EtOAc	BB	92	^119^
PBBS	reflux/2 h	EtOAc	BB	90	^119^
PBPS	reflux/2 h	EtOAc	BB	90	^119^

a HHP, high heat production; SF, solvent-free; HAS, homolytic aromatic substitution; [M], netal-catalyzed; NP, normal pressure; LA/LB, Lewis acid/base halogen-bonding interactions; RH, radical halogenation; BB, benzylic bromination.

### 3.1 Sustainable Sulfonation Strategies

Sulfonation is the most common method for introducing ion-conductive groups into a polymer to create a cation exchange membrane. However, conventional sulfonation methods typically involve the use of concentrated H_2_SO_4_ or HSO_3_Cl as sulfonating agents, which first dissociate into SO_3_ and their remaining components. In the next step, sulfur binds to the nucleophilic carbon, forming the sulfonated product while generating hazardous acid waste that poses significant environmental risks. Additionally, both acids are highly corrosive and dangerous to handle, making the process time-consuming and less cost-efficient.^50^ Furthermore, the properties of ion exchange membranes (IEMs) depend on the degree of functionalization, specifically the degree of sulfonation (DS), which directly influences the membrane’s proton exchange capacity. The DS can be controlled by adjusting the reaction temperature (−20 to 300 °C), reaction time, and the concentration of sulfonating agents.^103,104^

An alternative sulfonation method involves the direct use of SO_3_, offering advantages such as the elimination of acid waste, a fast reaction rate, and no corrosion of equipment. However, this approach also has drawbacks, including high heat generation and a gradual increase in viscosity over time when using liquid SO_3_.^105^ To prevent explosions caused by excessive heat production, sulfonation with SO_3_ can be carried out in a green solvent, allowing for better reaction control, while the choice of solvent should carefully consider the reaction byproducts and their potential hazards.^106^ The sulfonation of hydrocarbons and aromatic molecules can be carried out in water as a solvent using K_2_S_2_O_5_ as a green sulfonation agent under mild conditions, such as room temperature and ambient pressure. Kanbua et al.^107^ reported a sustainable synthesis of cellulose-based membranes by first oxidizing cellulose through gamma irradiation, followed by sulfonation in deionized water at room temperature under continuous stirring for 72 h. Han et al.^108^ demonstrated the development of a homolytic radical sulfonation method utilizing K_2_S_2_O_5_ or K_2_SO_3_ in combination with Mn­(OAc)_3_·2H_2_O and 1,1,1,3,3,3-hexafluoroisopropanol (HFIP). This innovative approach offers several advantages over traditional electrophilic sulfonation, including milder reaction conditions, compatibility with acid-labile protecting groups, the absence of waste acid production, and selective reactivity for specific structures. However, a notable drawback of this method is the reliance on fluorinated solvents such as HFIP. All the studies presented report the use of electrophilic sulfur trioxide derivatives (SO_3_) or SO_3_ complexes as sulfonating agents. The reactivity of these reagents depends on the presence of good leaving groups or their ability to form sulfonium ions. Additionally, sulfonation can also be carried out using nucleophilic agents, such as hydrogen sulfites (HSO_3_^–^) or sulfur dioxide (SO_2_). The nucleophilic substitution reaction typically occurs with halogenated substrates, while nucleophilic addition reactions can also take place with aldehydes or ketones.^103^ Zhang et al.^109^ presented a sustainable approach for synthesizing aryl and alkyl sulfonic acids using halides and thiourea dioxide (CH_4_N_2_O_2_S) as an eco-friendly and easily handled SO_2_ surrogate, with air as a green oxidant. This method employs a one-step strategy under mild conditions, utilizing a palladium-based catalyst (PdCl_2_(dppf)), which has been reported to work with aryl iodides and alkyl bromides. However, the drawbacks of this method include the high cost of the catalyst and the need for additional reagents to enhance the solubility properties of sulfur dioxide salts.

### 3.2 Sustainable Bromination Strategies

Bromination of polymers plays an important role in IEMs synthesis, not as a direct functionalization step but as a crucial pretreatment that enables further chemical modification. Unlike sulfonation, which introduces ionic groups directly, bromination installs reactive benzylic or aromatic bromine sites on the polymer backbone. These sites can then undergo nucleophilic substitution to introduce functional ionic groups, most commonly through quaternization with amines. The modification of IEMs through bromination thus represents a common method for introducing functional groups or reactive sites, enabling subsequent steps that impart ion exchange capability. However, bromination using molecular bromine (Br_2_) should be avoided, as it is hazardous, toxic to both the environment and human health, and difficult to handle. Alternatively, bromination with *in situ* synthesized bromine offers a safer approach, allowing for up to 100% atom efficiency while minimizing hazardous waste. Rahu and Järv^110^ reported a solvent-free synthesis of molecular bromine and its application for *in situ* bromination. The authors have demonstrated that molecular bromine can be generated from a mixture of solid KNO_3_ and gaseous HBr, as well as from a combination of solid NaBr, KNO_3_, and gaseous HCl. The produced bromine was then successfully used for the *in situ* bromination of *N*-phenylacetamide (acetanilide) and other aromatic molecules, following the electrophilic aromatic substitution mechanism. Additionally, the rate of bromine formation in this approach can be effectively controlled by adjusting the amount of KNO_3_ in the reaction mixture, enabling control over the reaction kinetics. This solvent-free method offers several advantages, including simplified product purification, minimal byproduct formation, the absence of solvent waste, and operation at ambient temperature, making it a more sustainable and efficient approach to bromination.

*N*-Bromosuccinimide (NBS) is another widely used agent in Wohl–Ziegler bromination reactions, which has lower toxicity, is easier to handle, and is able to operate under milder reaction conditions compared to Br_2_.^110–112^ It operates via a radical mechanism, generating a steady, low concentration of Br_2_ and HBr in the reaction medium, which enhances selectivity and minimizes side reactions, while to activate the reaction either a radical initiator or light irradiation is required.^113^ However, NBS still poses certain drawbacks, including its harmful nature, poor thermal stability, limited solubility, and low atom economy as it is used in 1:1 molar proportion. It is typically used with toxic, carcinogenic, and ozone-depleting chlorinated solvents like carbon tetrachloride (CCl_4_) due to its incompatibility with many other solvents (e.g., THF, toluene^114^).^115^ This reliance on hazardous solvents poses environmental concerns and limits the sustainability of the process, necessitating the exploration of greener alternatives. Marcos et al.^116^ presented a greener bromination approach using visible light to initiate the radical cascade, employing environmentally friendly acetonitrile as a solvent and avoiding the use of radical initiators.^117^ This method successfully achieved the bromination of 4-methylbenzoic acid at the methyl group, converting it into 4-bromomethylbenzoic acid at 55 °C in just 15 min.

De Almeida et al.^118^ demonstrated the bromination of alkylarenes using tribromoisocyanuric acid (TBCA) as a brominating agent under reflux in ethyl acetate (EtOAc), without requiring any catalysts or light irradiation. TBCA offers advantages over NBS, as it can transfer three bromine atoms to the substrate. Consequently, the stoichiometry of reactions involving TBCA is often 3:1 (substrate/TBCA). The study achieved 100% conversion within 6 h, yielding 84% of the 1-bromethylbenzene from ethylbenzene. Analogously, Ghorbani-Vaghei et al.^119^ investigated the bromination of benzylic positions using *N*,*N*,*N*′,*N*′-tetrabromobenzene-1,3-disulfonamide (TBBDA), poly­(*N*,*N*′-dibromo-*N*,*N*′-(ethylene)­benzene-1,3-disulfonamide (PBBS), and poly­(*N*,*N*′-dibromo-*N*,*N*′-(1,3-phenylene)­benzene-1,3-disulfonamide) (PBPS) as brominating agents. These reagents facilitate the selective transfer of multiple bromine atoms to the substrate, with TBBDA demonstrating the highest efficiency. In the bromination of ethylbenzene under reflux in EtOAc, TBBDA achieved a 92% yield in just 2 h, making the reaction three times faster than the method using TBCA. Figure 6 reports the molecular structure of the brominated compounds described above.

**Figure 6:**
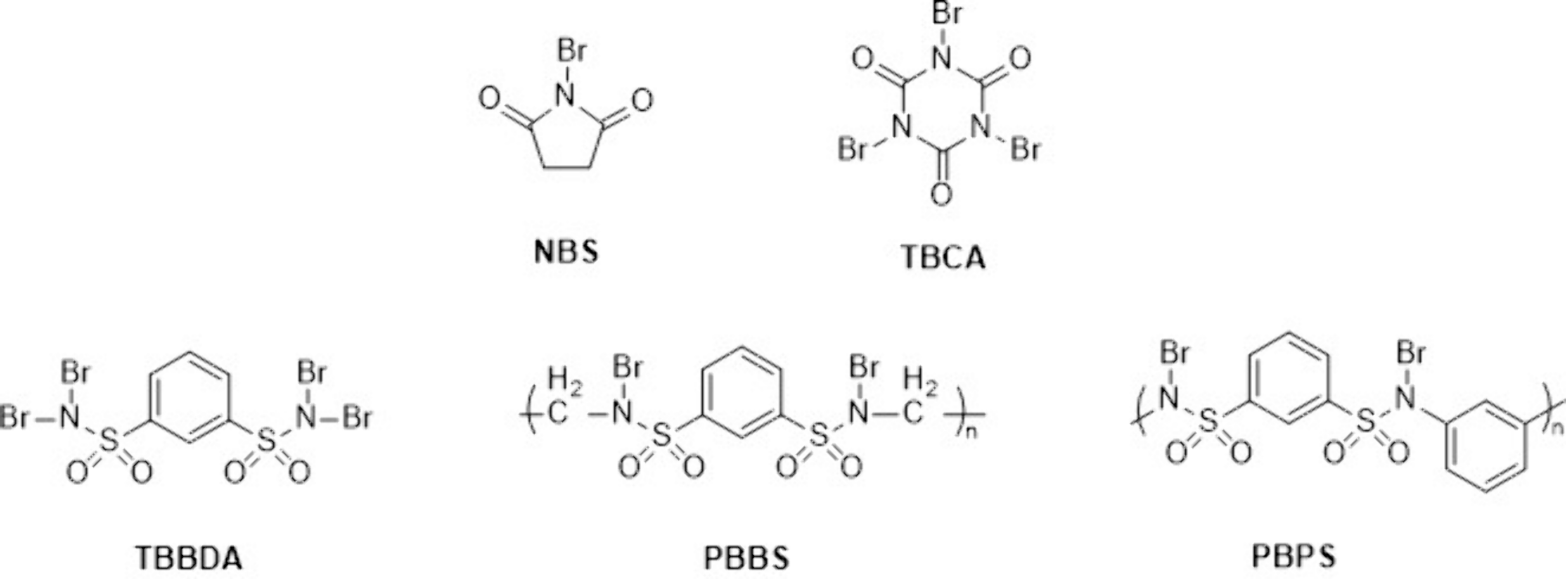
Schematic representation of the chemical structures of selected brominating agents.

Furthermore, bromination can be performed using various catalysts and brominating agents to minimize byproduct formation, eliminate the need for hazardous solvents, enhance selectivity, and to improve the overall reaction efficiency, while researchers continue to develop innovative and sustainable strategies for the functionalization of polymers.^111,120–122^ However, the main drawback of catalyzed bromination is the high cost of metal-based catalyst, along with its regeneration and recycling expenses, which significantly impact the overall membrane production cost. If production costs become too high, the process may no longer be commercially viable, ultimately negating the advantages of brominated membranes in practical applications.^123^

### 3.3 Membrane Fabrication

To utilize an IEM in a reaction cell, it must first be fabricated. Several methods exist to create a membrane from a polymer solution, each offering distinct advantages and resulting in different membrane properties. The most commonly used technique is casting, which can be performed under various conditions, either with or without a solvent, and at different temperature regimes.

When using the solvent casting method (see schematics reported in Figure 7), the polymer is first dissolved in a volatile solvent, typically a high-boiling-point polar aprotic solvent such as *N*-methyl-2-pyrrolidone (NMP), dimethyl sulfoxide (DMSO), dimethylacetamide (DMAc), or dimethylformamide (DMF). The polymer solution is then spread onto a substrate, enabling the solvent to gradually evaporate. However, strong interactions between the solvent and the sulfonic acid groups of the polymer can reduce the availability and mobility of protons, thereby affecting proton transport.^124,125^ He et al.^126^ reported that membranes cast using an ethanol/water solvent mixture exhibited higher proton conductivity, water uptake, and methanol permeability, while also benefiting from the sustainability of the solvent system. Alternatively, to commonly used solvent systems, ionic liquids can be considered as less toxic and more environmentally benign solvents for membrane preparation. Xing et al.^127^ reported, for the first time, the use of 1-butyl-3-methylimidazolium thiocyanate ([BMIM]­SCN) as a solvent for the fabrication of flat asymmetric membranes. They demonstrated that the ionic liquid could be recovered by distillation after membrane formation and reused in subsequent processes, thereby making the approach both greener and more economical.^128,129^ Controlling the evaporation rate (e.g., using an infrared lamp^130^ or oven^131^) is crucial for producing a dense and uniform membrane with excellent mechanical properties, while preventing defects such as cracking or uneven thickness. Additionally, a lower casting temperature was found to enhance proton conductivity, water uptake, and methanol permeability by promoting the aggregation of hydrophilic domains and increasing phase separation. The choice of substrate, whether porous or nonporous (e.g., a smooth glass surface), also plays a critical role in determining the final surface properties of the membrane.

**Figure 7:**
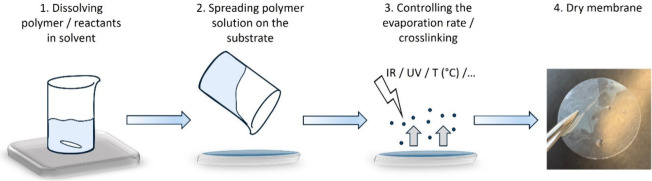
Membrane fabrication process using the solvent casting method.

In phase inversion casting, the polymer solution undergoes phase separation, which can occur through three main mechanisms: Thermally induced phase separation (TIPS), vapor-induced phase separation (VIPS), or nonsolvent-induced phase separation (NIPS).^50^ This technique allows the use of green solvents and environmentally friendly approaches and is widely applied to create membranes with controlled porosity and high ionic conductivity, making it suitable for a broad range of applications, including their use in PEC cells.^132^ However, both the solvent casting method and phase inversion casting often require the use of organic solvents, which cannot be avoided and can have harmful effects on the environment and human health, as well as increase production costs. An alternative solvent-free membrane fabrication method is the extrusion melt process, in which a polymer melt is extruded into a cooler atmosphere, inducing a phase transition that produces a dense and isotropic membrane. Ma et al.^132^ introduced a method for AEM fabrication through the hot-pressing of polyelectrolyte complexes, resulting in saloplastics with minimal waste production. This approach provides membranes with high long-term stability under extreme alkaline and acidic conditions, as well as excellent selectivity for monovalent ions.

In recent decades, numerous studies have explored sustainable synthesis methods for the functionalization and fabrication of IEMs, focusing on innovative and environmentally responsible approaches. The primary challenge lies in balancing ecological sustainability with maintaining high membrane performance. Key strategies include minimizing toxic waste and byproduct formation, replacing hazardous solvents with greener alternatives, and exploring solvent-free synthesis. Additionally, optimizing reaction parameters such as catalyst selection temperature, pressure, reagent concentration, and reaction time can enhance efficiency and reduce environmental impact. These advancements contribute to the development of safer and more sustainable ion exchange membranes for various applications.

The membranes discussed above are self-standing, thin, and dense films, which is essential for their application in PEC cells. In this context, they serve as physical separators of reaction products, preventing mixing and thereby ensuring high selectivity and stability of the system. At the same time, their dense, ion-conducting structure enables efficient charge exchange between the cell compartments. Their thinness minimizes resistance losses while maintaining sufficient mechanical integrity, making them particularly well-suited for PEC operation.

It may also be valuable to explore the potential of self-assembled nanostructured membranes or those incorporating proton-conducting channels, as they could provide alternative design strategies. However, such approaches lie beyond the scope of this review.^133,134^

## 4 Membrane Characterization

The performance of IEMs in (photo)­electrochemical applications is largely determined by key physicochemical, electrochemical, mechanical, and morphological properties. To study and evaluate these properties, various characterization techniques can be applied, each providing insights into different aspects of membrane behavior. Among these techniques, thermogravimetric analysis (TGA)^135,136^ and differential scanning calorimetry (DSC)^137^ provide insights into the thermal stability of the polymer. Cyclic voltammetry (CV)^138^ is used to assess electrochemical stability, while permeability tests help determine gas permeability. Additionally, chemical stability tests allow the study of oxidative resistance and degradation behavior. Morphological characterization is particularly important for understanding membrane performance and stability in PEC cells. Scanning electron microscopy (SEM) has been widely used to examine membrane surfaces. SEM provides both qualitative observations of surface features and quantitative information such as pore size, pore size distribution, pore shape, porosity, surface roughness, and fouling, which influence ion transport and interfacial interactions.^139^ Mechanical properties, including tensile strength, elongation at break, and Young’s modulus, provide insight into membrane robustness, flexibility, and stiffness, while additional tests such as tear resistance, puncture resistance, and dynamic mechanical analysis (DMA) evaluate durability under operational stress.^140^ Together, these properties ensure structural integrity and stable ion transport during swelling, hydration cycles, and mechanical stresses in PEC operation.

While standard techniques such as ion exchange capacity (IEC) and water uptake (WU), which are briefly described in this chapter, provide fundamental information on the density of ionic groups and the hydration behavior of the membrane, both of which directly influence ion transport and overall conductivity, more advanced characterization methods are required for PEC applications, where membranes are exposed to light, bias, and reactive electrolytes. Electrochemical impedance spectroscopy (EIS)^141^ is a powerful technique for probing ionic conductivity, charge-transfer resistance, and transport pathways within the membrane under relevant operating conditions, providing quantitative insight into its electrochemical performance. Complementarily, ambient-pressure hard X-ray photoelectron spectroscopy (AP-HAXPES)^142^ enables detailed analysis of the chemical composition and oxidation states at the membrane surface and near-surface regions under near-operando conditions, revealing potential degradation mechanisms or interfacial chemical changes during PEC operation. By integrating both standard and advanced techniques, a comprehensive understanding of the membrane’s structural, chemical, and electrochemical properties can be achieved, supporting the rational design and optimization of IEMs for high-performance PEC devices.

### 4.1 ion exchange Capacity

The ion exchange capacity (IEC) refers to a material’s ability to exchange ions with a surrounding solution and is typically expressed in milliequivalents per gram (mequiv/g). The IEC value is influenced by the number and type of functional groups or active sites available within the material, which facilitate the ion exchange process. The IEC can be determined using methods such as acid–base or back-titration, where different titrant solutions are selected based on the type of membrane being analyzed. Thus, to determine the IEC of a CEM, the dried membrane should first be immersed in a HCl solution for up to 24 h and then washed with deionized water until the pH is neutral. Next, the membrane is immersed in a saturated solution of potassium chloride (KCl) or sodium chloride (NaCl) to replace the H^+^ ions with K^+^ or Na^+^ cations. Finally, the resulting solution containing the replaced protons is titerd using a NaOH solution with a known concentration and an indicator such as phenolphthalein.^143^ Analogously, the determination of IEC of AEM requires first the displacement of the anions and then the titration in the presence of an indicator.^144^ The IEC (in mequiv/g) can be calculated by the following equation:

1 $$\mathrm{IEC}=\frac{V_{\mathrm{titrant}}\cdot c_{\mathrm{titrant}}}{m_{\mathrm{dry}}}$$

where *V*_titrant_ is the titrant volume needed to neutralize the solution, *c*_titrant_ is the concentration of the used titrant, and *m*_dry_ is the mass of the dry sample. Although titration is the most commonly used method for determining IEC in the literature, several other techniques are available, including nuclear magnetic resonance (NMR) spectroscopy,^145,146^ Fourier-transform infrared (FTIR) spectroscopy,^147^ thermogravimetric analysis (TGA), energy-dispersive X-ray (EDX) spectroscopy, conductivity measurements, and elemental analysis (EA).^148^ Moukheiber et al.^149^ demonstrated in 2012 that titration is one of the methods with the highest error. Among all techniques, titration and FTIR exhibit the greatest uncertainties, while NMR is recommended as the most reliable method whenever possible.^150^

### 4.2 Water Uptake

Water uptake (WU) refers to a membrane’s ability to absorb and retain water, which is influenced by its chemical composition, pore structure, and the hydrophilicity of its functional groups. It is determined by comparing the membrane’s mass after being soaked in water or a solution for an extended period with its dry mass after being dried in a vacuum oven until a constant weight is reached and can be calculated as follows:

2 $$\mathrm{WU}=\frac{m_{\mathrm{wet}}-m_{\mathrm{dry}}}{m_{\mathrm{dry}}}\times 100\%$$

where *m*_wet_ and *m*_dry_ are the masses of the wet and dry membranes, respectively. The change in membrane length due to swelling is quantified by the swelling ratio, which can be calculated using an analogous formula as follows:

3 $$\mathrm{swelling}\;\mathrm{ratio}=\frac{L_{\mathrm{wet}}-L_{\mathrm{dry}}}{L_{\mathrm{dry}}}\times 100\%$$

where *L*_wet_ and *L*_dry_ are the lengths before and after soaking in water, respectively. A higher WU generally enhances ion conductivity by facilitating ion transport through hydrated pathways but may also reduce mechanical stability, leading to excessive swelling or degradation.

### 4.3 Electrochemical Impedance Spectroscopy

Electrochemical impedance spectroscopy (EIS) is a technique used to investigate the electrical characteristics of conductive materials and their interfaces. It finds applications across various fields, including materials science, corrosion studies, semiconductors, conductive polymers, ceramics, coatings, energy storage, and solid-state technologies. In EIS, the potential applied to the electrochemical system is modulated with small sinusoidal perturbation (E_AC_), resulting in an alternating current (i_AC_). The amplitude and phase angle of this current provide valuable information about the system’s electrochemical properties, including electrical resistance in the current path, interfacial capacitance, mass transfer of reactants, and kinetics of all relevant heterogeneous and homogeneous reactions. EIS also enables the investigation of charge transfer and storage processes, which is crucial for the characterization of IEMs. By varying the frequency of the sinusoidal perturbation, typically between 10^–4^ to 10^6^ Hz, the system’s response at multiple time scales is analyzed, allowing for a comprehensive analysis of the electrochemical system’s behavior. The electrochemical impedance *Z* is a function of the perturbation angular frequency ω = 2π*f*, where *f* represents the conventional frequency. *Z*(ω) can be expressed as

4 $$Z\left(\omega \right)=\frac{Ẽ\left(\omega \right)}{Ĩ\left(\omega \right)}=\left|\frac{Ẽ\left(\omega \right)}{Ĩ\left(\omega \right)}\right|\left(\cos\varphi \left(\omega \right)+j\;\sin\varphi \left(\omega \right)\right)$$

where *Ẽ* and *Ĩ* are rotating vectors or phasors, which are complex time-invariant numbers that represent the amplitude (length of vectors) and phase of a sinusoidal function. As shown in eq 4, the relation between the potential and current signals can be represented as two sinusoidal periodic waves (Figure 8).^151^ Both signals oscillate at the same frequency (ω) and amplitude since one induces the other. However, there is a time shift between the signals, a phase-angle shift (φ), which can range from −90° to 90°. The phasors *Ẽ* and *Î* can be represented as functions of time by

5 $${Ẽ}_{t}=E_{0}\;\sin\left(\omega t\right)$$

and

6 $${Ĩ}_{t}=I_{0}\;\sin\left(\omega t+\varphi \right)$$

where *Ẽ*_*t*_ is the AC component of the potential at time *t*, *E*_0_ is the amplitude of the signal, *Ĩ*_*t*_ is the instantaneous current at time *t*, and *I*_0_ is the amplitude of the current.

**Figure 8:**
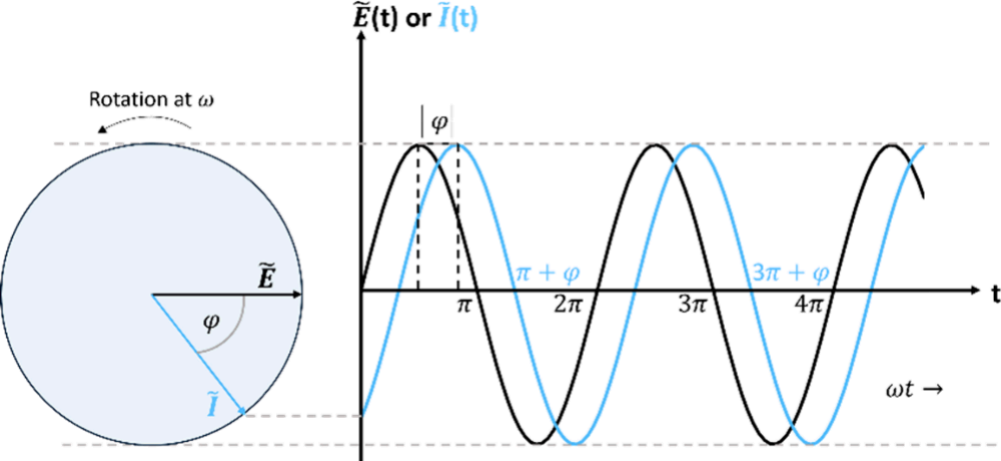
Sinusoidal signals for *Ẽ* and *Î* phasors.

Since the current and voltage phasors rotate at the same frequency, their relationship to each other and the phase angle remain constant. Thus, they can be simply plotted as a single impedance vector. This vector can be represented using complex notation, where the component along the *x* axis is real, and the component along the *y* axis is imaginary, multiplied by j = $\sqrt{-1}$ .

7 $$Z\left(\omega \right)=Z^{\prime }+jZ^{\prime \prime }$$

where *Z*′ is the real part and *Z*″ the imaginary part of the impedance.^152,153^ Introducing complex notation helps keep the vector components straight. Despite their mathematical labels as “real” or “imaginary”, both components can be measured through the phase angle. The impedance serves as a generalized form of resistance and the phase angle indicates the relationship between capacitive and resistive components in an electrical circuit. For a purely resistive circuit, φ = 0; for a purely capacitive circuit, it is φ = π/2, and for circuits containing both elements, the phase angle falls between these values, depending on the balance of resistance and capacitance. Note that the phase angle can also become negative, indicating an inductive response (φ = −π/2 for a pure inductor). Connecting cables or wires can cause an inductive response at high frequencies, but we will ignore this effect in the rest of the discussion. When the amplitude of the applied alternating current (AC) signal is kept sufficiently low (typically 5–20 mV_peak‑to‑peak_), the system behaves in a manner where the input and output signals retain the same frequency, an indication that the system is linear and time-invariant (LTI) as defined in control theory. Under these conditions, the complex impedance can be modeled by an equivalent network (the so-called electrical equivalent circuit, or EEC) composed of basic circuit elements like resistors, capacitors, and Warburg components. The combination of elements describes how the polymer material responds to the external perturbation and thus captures the underlying electrochemical and relaxation processes within the polymer.

The graphical representation of impedance data in EIS is often presented as a Nyquist plot, which visualizes eq 4 by plotting the imaginary part of impedance on the *y* axis against the real part on the *x* axis (−*Z*″ versus *Z*′) (Figure 9). For EIS measurements of charge transfer, the EEC consists of parallel elements representing contributions from the Faradaic process and double-layer charging. The double-layer capacitance behaves nearly as a pure capacitor and is represented by element *C*_dl_. The faradaic process, which involves charge transfer, is represented as a general impedance that includes charge transfer resistance *R*_ct_ and the Warburg element *Z*_W_, accounting for diffusion effects.

**Figure 9:**
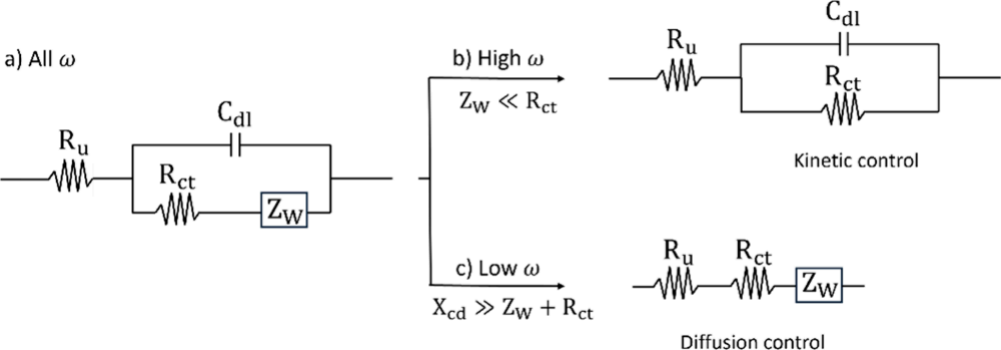
Randles equivalent electrical circuit (EEC) for charge transfer measurements. *R*_u_ is the uncompensated resistance, *R*_ct_ is the charge-transfer resistance, *C*_dl_ is the differential capacitance of the double layer, and *Z*_W_ is the Warburg element.

As illustrated in Figure 10, at high frequencies, the Nyquist plot begins at the origin or at a small offset on the *x* axis due to the electrolyte and membrane bulk resistance (*R*_u_). The semicircle observed in the mid- to high-frequency range corresponds to the charge transfer process, with its diameter representing the charge transfer resistance. At lower frequencies, a linear region appears, representing Warburg impedance, which accounts for mass transport limitations. This characteristic shape provides valuable insights into the electrochemical system.

**Figure 10:**
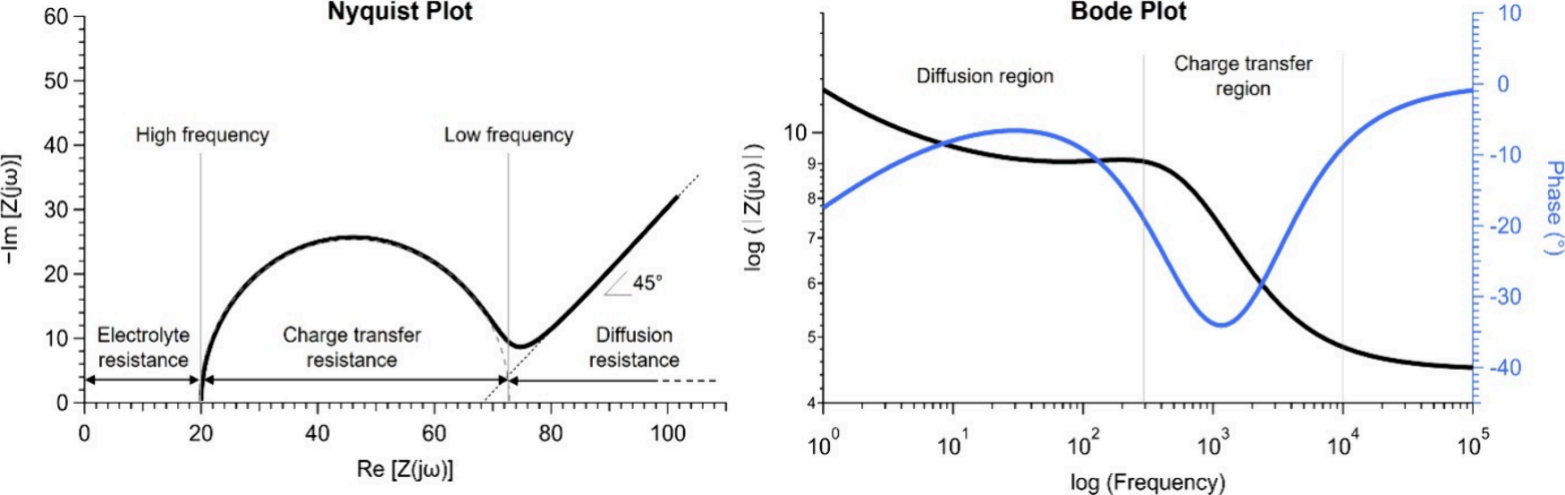
Nyquist and Bode plots over a frequency range of 1 Hz–100 kHz. The data have been obtained simulating the Randles circuit reported in Figure 9 for semi-infinite linear diffusion, using the following parameters: *R*_u_ = 20 Ω, *R*_ct_ = 53 Ω, *C*_dl_ = 10 μF, and *A*_W_ = 80 Ω·s^–1/2^.

Another common graphical representation is the Bode plot, which consists of two plots in one (Figure 10). It displays both the magnitude of impedance and its phase angle as a function of frequency. This makes the Bode plot particularly useful for analyzing a system’s resistive and reactive components across a wide frequency range. The *x* axis represents the logarithmic scale of frequency, with one *y* axis showing the logarithm of impedance and the other *y* axis displaying the phase shift.

Using impedance measurement data, the proton conductivity of a CEM can be determined using the following equation:

8 $$\sigma =\frac{L}{R\cdot A}$$

where *L* is the thickness (cm), *A* is the face area (cm^2^) of the membrane, and *R* is the resistance of the membrane (the *Z*′ value when −*Z*″ = 0 ohm). A practical way to determine the membrane resistance and distinguish it from the electrolyte resistance is to perform impedance measurements both with and without the membrane. By comparing the two, the contribution of each component to the total resistance can be evaluated. To ensure proper proton conductivity measurements, the membrane must first undergo activation to exchange counterions and remove impurities. This process involves thorough cleaning with water followed by soaking in an acid solution for an extended period of time. This activation step enhances the membrane’s ionic conductivity and overall performance in electrochemical applications.

Another possibility to represent EIS data for membranes is through a transmission line model (TLM), which was first introduced in 1963 by De Levie.^153^ This model allows for the mathematical interpretation of the penetration depth of an alternating voltage signal, enabling the determination of characteristic frequencies associated with different membrane layers. It treats resistance and capacitance as distributed parameters along a heterogeneous, porous, or structured membrane, accounting for variations in conductivity. The TLM is particularly useful for describing the effects of diffusion, electromigration, and double-layer phenomena, making it a valuable tool for analyzing complex ion transport mechanisms in membranes.^154,151^ The TLM can be represented as a series of segments, each incorporating ionic resistance, double-layer capacitance, and charge transfer resistance.^155,156,56^ Typically, TLM includes *n* segments, where each segment accounts for the spatial variation of ion transport and electrochemical interactions within the membrane structure. Figure 11 illustrates a possible TLM configuration.^155,157^

**Figure 11:**
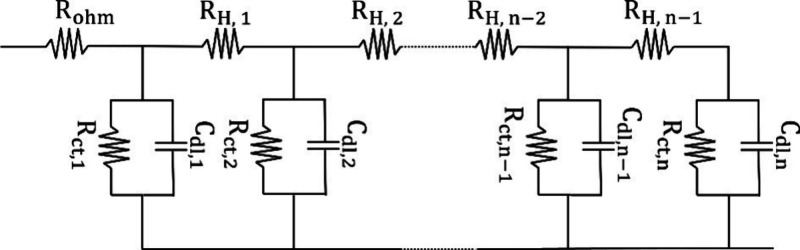
Transmission line model with ion-transfer resistance (*R*_H_), double layer capacitor (*C*_dl_), and the charge transfer resistance (*R*_ct_). Adapted with permission from ref 157. Copyright 2018 Hydrogen Energy Publications LLC.

Using the TLM, Qaiser^158^ demonstrated that the transport processes in mixed cellulose ester-polyaniline composite membranes are strongly influenced by both the content and the deposition site of polyaniline within the membrane structure. Various electronic and ionic diffusion processes were characterized by evaluating characteristic frequencies associated with these transfer processes. Additionally, it was shown that different polymerization techniques significantly affect the charge transport properties of the composite membrane. Thus, this modeling approach provides insights into the membrane composition and morphology. Jing and Chaplin^154^ presented a study using the TLM to spatially characterize membrane fouling and the regeneration process. By analyzing the membrane at different frequencies, they provided insights into distinct membrane regions, including the outer surface, active layer, and support layer. Additionally, their research examined fouling mechanisms, such as monolayer adsorption, demonstrating the effectiveness of EIS in analyzing membrane behavior and performance.

To illustrate how EIS measurements on membranes can be performed, we briefly discuss the testing apparatus illustrated in Figure 12. This home-built setup was designed and constructed to evaluate the interplay between the membrane’s chemical composition, thickness, electrolyte composition, and reaction environment on the overall performance (activity, selectivity) of the (photo)­electrolyzer. The IEM under investigation is positioned between two flow chambers, each equipped with independent inlets and outlets for fluid control, along with two ports for accommodating electrodes. The cell was controlled by a potentiostat in combination with a frequency response analyzer (BioLogic VSP 300). The system enables measurement of DC resistance, AC impedance, and voltage losses under constant current conditions (galvanostatic mode), utilizing a four-terminal (or four-point probe) method.^159^ Also known as Kelvin sensing, this technique uses separate electrode pairs for current supply and voltage sensing, which significantly enhances measurement accuracy by eliminating the influence of lead and contact resistances. In this configuration, current is delivered through a pair of platinum rods (current or power electrodes), creating a voltage drop across the membrane. A second, independent pair of voltage-sensing reference electrodes is positioned in close proximity to the membrane to detect the resulting voltage drop. Due to the extremely high input impedance (on the order of 10^12^–10^13^ Ω) of the potentiostat’s reference and sense inputs, virtually no current flows through the sensing electrodes. This ensures that the measured voltage accurately reflects the membrane’s true impedance, free from interference by contact or wire resistances.^5^ Our system supports AC impedance measurements over a wide frequency range, from 1 MHz to 1 mHz, covering nine decades.

**Figure 12:**
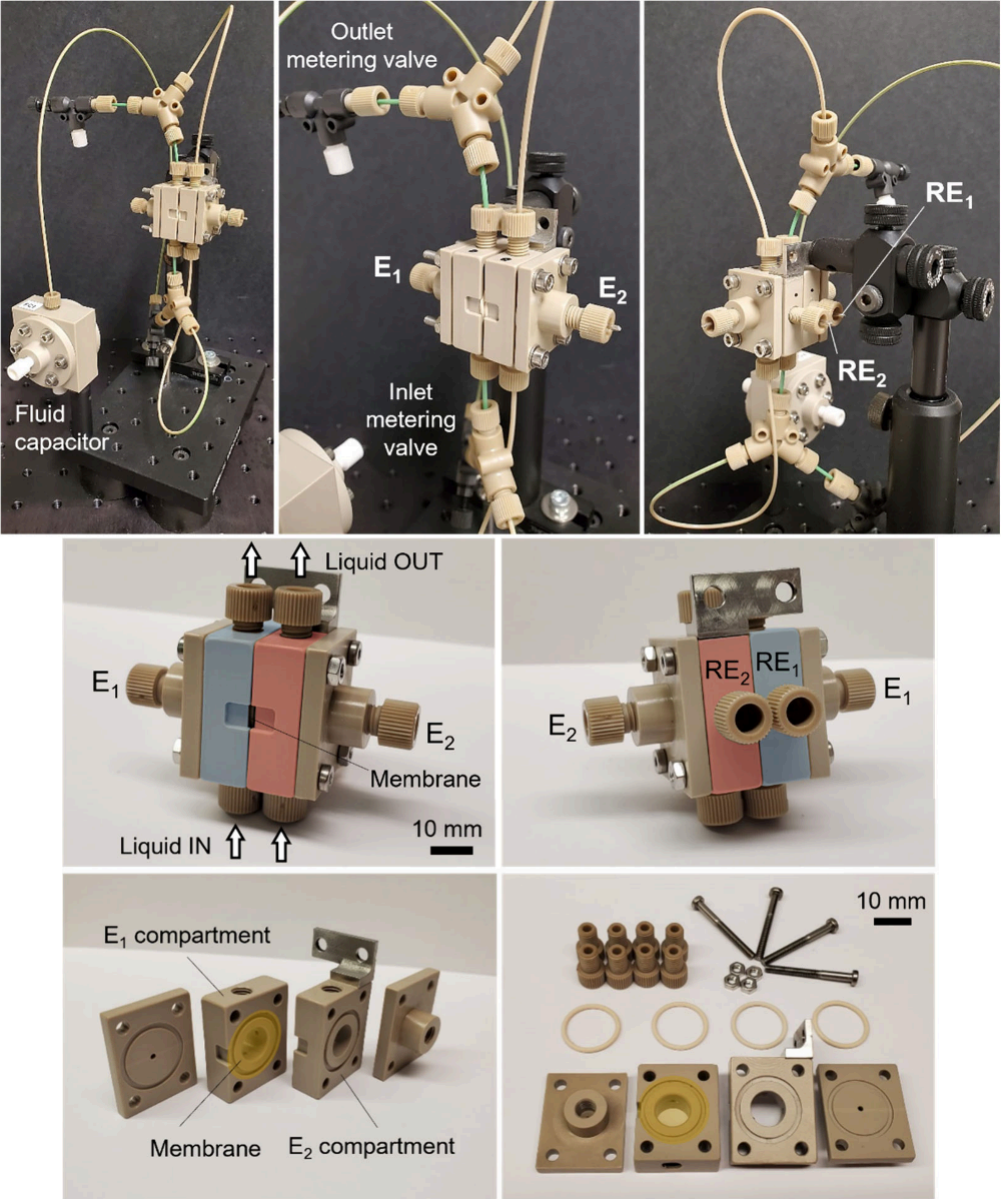
Customized flow device developed for the investigation of DC resistance, AC impedance, and voltage loss at fixed current density across the membrane under test using a four-terminal sensing technique.

It is noteworthy mentioning that interpreting impedance data through the analysis of the EEC remains a highly expert-driven task, often limited by subjective model selection and time-intensive fitting procedures. To address these limitations, a recent work by Albakri et al. have introduced a convolutional neural network (CNN)-based model that predicts EEC topologies directly from experimental impedance spectra, reducing user bias and enabling faster, more objective analysis.^159^ This machine learning (ML)-assisted framework achieved a top-5 classification accuracy of ∼80% and demonstrated the ability to extrapolate data at low frequencies where experimental measurements are often unreliable but crucial for identifying diffusion-related processes like Warburg impedance. When validated on real BDS data from membranes such as Nafion N115 and Fumasep FAA-3-PK-75, the CNN outperformed conventional fully connected networks, owing to its capacity to capture and process temporal features in the spectral data.^159^ Importantly, the constrained search space and regularization strategies embedded in the model help mitigate the issue of nonidentifiability in circuit modeling, ensuring physically meaningful parameter extraction. The integration of sparse regression methods like Lasso for circuit parameter fitting could further refine the model by promoting interpretable sparsity and automatic topology selection. Additionally, incorporating the frequency vector as an input and generating training data over varied spectral conditions can enhance the generalizability of the model to real-world measurements. As a result, the overlap of ML techniques with electrochemical characterization offers a powerful, scalable strategy for accelerating the discovery and optimization of advanced polymer membrane materials.

To summarize, EIS measurements enable the understanding of ion transport and proton conductivity in IEMs and is a widely used technique for membrane resistance measurements.^56^ The conductivity or resistance of the IEM can be determined without interference from the electrolyte by directly contacting an activated IEM between two electrodes and applying a potential.^160^ In addition, EIS measurements can be conducted in a (flow) electrochemical cell in contact with liquid electrolytes, providing valuable insights into the membrane’s conduction properties under real operating conditions. This approach enables for the evaluation of ion mobility, charge transfer resistance, and overall membrane performance in practical applications.

### 4.4 Ambient-Pressure Hard X-ray Photoelectron Spectroscopy (AP-HAXPES)

Ambient pressure hard X-ray photoelectron spectroscopy (AP-HAXPES) represents a technique used to analyze the chemical composition and electronic states of materials. AP-HAXPES enables direct insights into the physical and chemical properties of polymer membranes under realistic conditions at relatively high pressures, up to and above the vapor pressure of water at room temperature (about 25 mbar). This offers a significant advantage over conventional soft X-ray photoelectron spectroscopy (XPS) (*h*ν ∼ 200–1500 eV), which is typically performed in ultrahigh vacuum (UHV, ≤ 10^–10^ mbar) to minimize the electron scattering by gaseous molecules.^161^ The use of high-energy X-rays provides detailed information on the surface and near-surface composition (down to 20–30 nm), while also allowing the characterization of the oxidation states and chemical environment of the elements present in the sample under investigation.^142^ Operating under ambient conditions is quite important for understanding the electrochemical behavior of IEMs, since properties like mechanical and chemical stability, as well as ionic conductivity, are strongly influenced by the level of hydration and the chemical environment in which the membranes function. Furthermore, AP-HAXPES enables the direct determination of local built-in electrical potentials by measuring the kinetic energy shift of detected photoelectrons. Importantly, AP-HAXPES enables the study of the surface on the triple-phase boundary formed on a solid/liquid/gas interface.^162^ Figure 13 reports a digital photograph of the analysis chamber and of a customized flow cell for membrane research available at the Spectroscopic Analysis with Tender X-rays (SpAnTeX) end-station at the BESSY II synchrotron facility in Berlin, Germany.^163^

**Figure 13:**
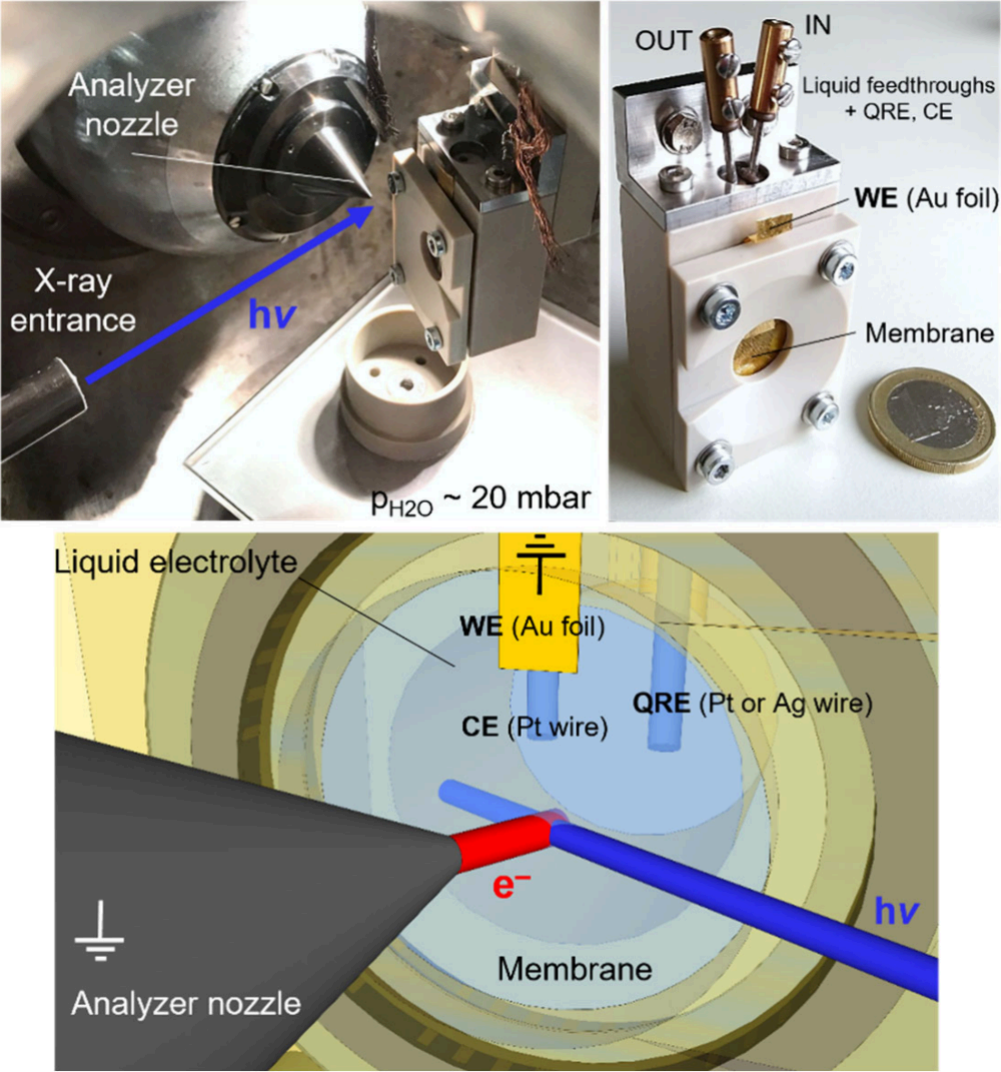
Analysis chamber and customized flow cell for membrane research available at the Spectroscopic Analysis with Tender X-rays (SpAnTeX) end-station, BESSY II synchrotron facility (Germany). The main analytics of the end-station are AP-HAXPES and AP-XAS (X-ray absorption spectroscopy). Adapted from ref 142. CC BY 3.0.

AP-HAXPES operates by utilizing synchrotron radiation (SR) to generate high-energy X-rays, typically ranging from 2–10 keV.^164^ These X-rays irradiate the material, causing the ejection of photoelectrons from the core levels of atoms, while the kinetic energy of the emitted electrons is analyzed. The intensity of a photoelectron peak is influenced by several factors, such as incident X-ray flux, the area of the illuminated sample seen by analyzer, the solid acceptance angle of the analyzer, the density of atoms, the differential photoelectric cross section, the overall detection efficiency, and the photoelectron mean free path (eIMFP, or *λ*_e_(*E*_kin_)). The eIMFP depends on the kinetic energy and the composition/electronic properties of the sample under investigation, and describes the average of effective depth from which photoelectrons can escape without significant energy loss due to inelastic electron scattering.^165–167^ The number of photoemitted electrons *N* that are ejected from the sample is related to the depth at which the emission takes place by a Beer–Lambert type of law:

9 $$N=N_{d}\;\exp\left(-\frac{d}{\lambda_{e}}\right)$$

where *N*_*d*_ is the number of electrons emitted at the depth *d* within the sample. This equation indicates that as the depth increases, fewer electrons will successfully escape the sample due to the inelastic scattering that occurs as they travel through the material. The information depth in XPS is typically defined as the distance from the sample surface at which 95% of the emitted electrons undergo inelastic scattering before they can reach the surface. Transforming eq 9 results in the following equations:

10 $$\frac{N}{N_{d}}=\exp\left(-\frac{d}{\lambda_{e}}\right)=0.05$$

11 $$-\frac{d}{\lambda_{e}}=\ln\left(0.05\right)$$

12 $$d∼3\lambda_{e}$$

The electron transmission by ambient pressure, *I*_*p*_/*I*_vac_, can be calculated as follows:

13 $$\frac{I_{p}}{I_{\mathrm{vac}}}=e^{-z/\lambda_{e}\left(E_{\mathrm{kin}}\right)}=e^{-\sigma pz/k_{B}T}$$

where *I*_*p*_ is the intensity under ambient pressure, *I*_vac_ is the intensity in vacuum, and *z* is the electron traveling length.

In HAXPES measurements, the bulk sensitivity is primarily influenced by the eIMFP of the electrons, which is affected by several factors. Fadley^168^ demonstrated, that at high-energy X-ray irradiation, the degree of surface or bulk sensitivity can vary by changing the electron exit angle θ_e_ due to more forward-peaked elastic electron scattering and a smaller effect from the inner potential at the surface. At higher energies in the range between 5 and 10 keV, the electron IMFP λ_e_(*E*_kin_) is expected to vary as (*E*_kin_)^0.50–0.75^, and the mean sensing depth corresponds to λ_e_ sin θ_e_, allowing the surface sensitivity to be controlled by adjusting the electron exit angle. Therefore, the highest bulk sensitivity is achieved close to normal emission, perpendicular to the surface (90°).^169^ Importantly, bulk sensitivity depends not only on the electron exit angle but also on the irradiation energy; measurements at 10 keV are more bulk-sensitive than at 1 keV, while the measurement depth is approximately 3 to 5 times larger. Additionally, the bulk sensitivity is affected by the inner potential V_0_ at the sample surface, which typically ranges from 5 to 25 eV and causes significant bending of the photoelectrons as they exit the surface. This refraction effect is more pronounced at grazing emission angles, which are close to the surface plane. Grazing angles are often used to enhance surface sensitivity in photoelectron spectroscopy because they decrease the electron escape depth, making the measurement more sensitive to surface characteristics. In the context of membrane research, AP-HAXPES measurements enable the identification of the ion exchange membrane (IEM) chemical composition at the surface and near-surface regions as reported in Figure 14a,b for commercially available Fumasep FAA-3-75 and Nafion N115 membranes. Moreover, this technique provides insight into the ion transport mechanisms, such as diffusion and electromigration, via the detection of specific ionic species and electrical potentials across the membrane. Furthermore, *in situ* investigations of IEMs using AP-HAXPES allows real-time monitoring of their physical and chemical properties under realistic working conditions. This includes monitoring the evolution of the membrane’s near-surface chemical composition while applying electrical potentials of different polarities across the membrane, offering valuable information on its performance and stability.^142,170,171^ Ralaiarisoa et al. employed AP-HAXPES to investigate two widely used commercial membranes, Nafion 115 (CEM) and Fumasep FAA-3-PK-75 (AEM), within a hybrid liquid/gas electrolyzer, a configuration increasingly relevant for (photo)­electrochemical CO_2_ reduction technologies.^142^ The experiments were conducted at water vapor pressures close to room temperature humidity conditions (15 to 20 mbar), using a model 1.0 M NaCl electrolyte. This enabled monitoring the near-surface chemical evolution of the membranes during polarization. The authors observed distinct ionic fingerprints: Na^+^ signals in Nafion and Cl^–^ in Fumasep, which clearly indicated ion out-diffusion at the open-circuit potential and under applied bias, largely independent of the bias polarity or magnitude as represented in Figure 14c,d. Here, it can be observed that while the spectral position of both C and Na 1s core levels shift as expected with the applied potential, their ratio does not exhibit any dependency upon the external bias.^142^ This behavior suggested that ion diffusion in such hybrid (photo)­electrolyzers is primarily governed by interactions with fixed charged groups and hydration dynamics, rather than by electromigration. Finite element simulations based on the Nernst–Planck equation, run at the same temporal and spatial resolution as the experiments, confirmed these observations and supported the conclusion that electrostatic and hydration effects dominate ion transport in these systems.^142^ Importantly, AP-HAXPES also enabled us to detect undesired polarization fields forming at the interface between the membrane and liquid electrolyte under specific biasing conditions. These fields emerged when the polarity of the applied potential matched the charge of the co-ions (those with the same charge as the fixed functional groups in the membrane), leading to co-ion accumulation at the interface.^142^ This accumulation caused a local drop in cell voltage, contributing to decreased device efficiency and selectivity.^172^ By identifying these interface-specific phenomena, this work demonstrated the utility of AP-HAXPES not only for chemical and electronic analysis, but also for uncovering interfacial charge effects that directly impact the performance of electrochemical devices.

**Figure 14:**
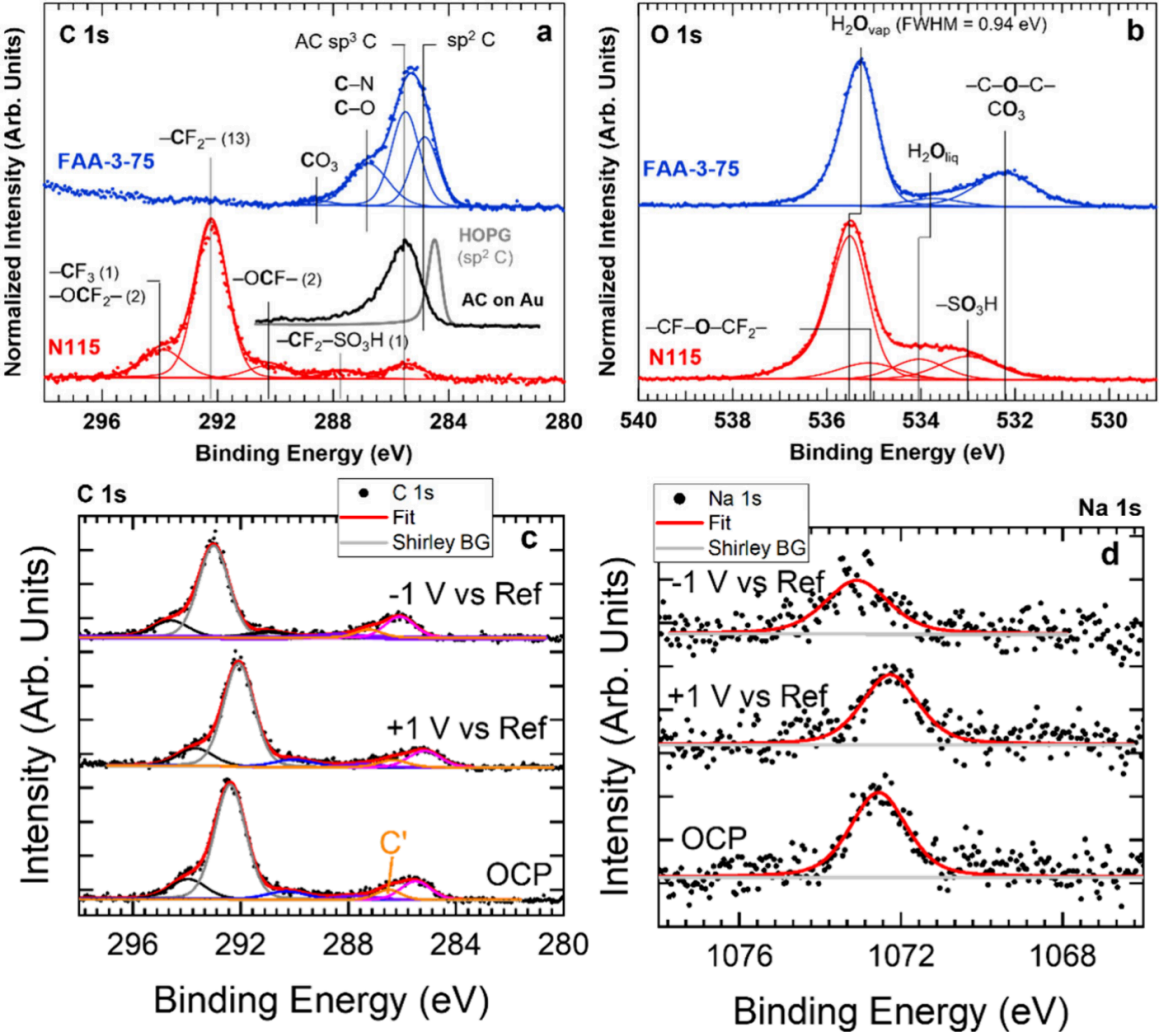
C 1s (a) and O 1s (b) core level spectra taken at the OCP on spectral references and on commercially available Fumasep FAA-3-75 and Nafion N115 membranes. C 1s (c) and Na 1s (d) core level spectra acquired on a Nafion N115 membrane in contact with a 1.0 M NaCl solution. The data were acquired under different applied potentials: half-cell OCP (+5 mV), +1 V, and −1 V. The AP-HAXPES measurements were conducted at a photon energy of 3000 eV, at room temperature (∼298 K) and at about 18 mbar of H_2_O. Adapted from ref 142. CC BY 3.0.

## 5 Impact of Membrane Properties on Device Performance

Until now, the performance of PEC devices has largely been assessed based on the optoelectronic properties of the photoabsorbers and the device architecture, where most energy losses typically occur.^172^ As a result, the contribution of the membrane to voltage losses has often been overlooked by the PEC community, partly due to the relatively low current densities achieved under nonconcentrated sunlight. However, with the development of novel polymers for cation exchange membranes—whose properties have yet to be systematically benchmarked—this aspect deserves renewed attention. To the best of our knowledge, such new polymers have not yet been explored in PEC devices, despite their successful application in systems such as redox flow batteries (RFBs). In both RFBs and PEC cells, membrane characteristics directly affect key performance metrics including efficiency, selectivity, and stability.

Tsehaye et al.^85^ investigated how membrane properties, such as ion exchange capacity, water uptake, membrane resistance, and thickness, affect the performance of aqueous organic redox flow batteries (AORFBs), highlighting the importance of understanding the correlations between these properties. For the cell cycling stability and performance test, an AORFB employing dimethyl viologen (MV) and *N*,*N*,*N*-2,2,6,6-heptamethylpiperidinyloxy-4-ammonium chloride (TMA-TEMPO) was chosen. Using four different AEMs based on poly­(*p*-phenylene oxide) with IECs varying between 1.5 and 2.8 mmol of Cl^–^·g^–1^, the study demonstrated that membranes with higher IEC exhibited increased water uptake, enhanced chloride ion mobility, and reduced membrane resistance. However, excessive water uptake negatively impacted cyclability during charge/discharge tests, due to crossover of active species across the membrane, leading to reduced capacity retention. On the other hand, membranes with low IEC exhibited reduced crossover and maintained high capacity retention after 100 consecutive charge/discharge cycles. In nonaqueous ORFBs, the membrane often swells as a result of absorbing the organic solvent.^173^ This swelling leads to an enlargement of the pore size, which reduces the membrane’s selectivity and negatively impacts the cell’s efficiency.^174^ Certainly, membrane resistance is generally influenced by both its thickness and ionic conductivity, with increased thickness and reduced conductivity resulting in higher resistance and diminished overall efficiency. Importantly, ionic conductivity is influenced by two key factors: The membrane’s water content and the operating temperature.^175^ In a PEC cell, the temperature can rise during illumination, which enhances ion conductivity, reduces internal resistance, and ultimately improves overall efficiency. Cell efficiency can be evaluated using polarization curves, which illustrate the voltage output of the cell as a function of current density and are directly influenced by membrane resistance. These curves help identify distinct regions associated with activation losses, ohmic losses, and mass transport limitations.^176^

Small et al.^177^ analyzed in detail the transport mechanism and capacity losses caused by crossover of species across five commercial AEMs during the operation of RFB based on MV/TEMPO. They considered the major transport mechanisms to be pressure-driven flow, solvent diffusion, diffusion of redox-active species, migration, and electroosmotic drag. The crossover of redox-active species was found to cause capacity losses of more than 50% of the theoretical capacity after just 100 cycles. Additionally, it was found that optimizing a membrane for one transport mechanism could inadvertently worsen its performance by facilitating another (undesired) mechanism. This highlights the complexity of membrane design for RFBs. Notably, membranes with the lowest diffusion coefficients for TEMPO and MV still exhibited high crossover fluxes, primarily due to electroosmotic drag rather than molecular diffusion. These findings underscore the need for a balanced design strategy that accounts for the interplay of multiple transport phenomena to minimize capacity fade and improve long-term battery performance. In summary, the optimal aqueous/nonaqueous ORFB performance can be achieved by minimizing cell resistance and reducing electrolyte crossover through a balance of ion conductivity and water/solvent uptake, while enabling high current density operation. In addition, membranes must exhibit excellent mechanical strength and chemical resistance to maintain long-term cycling stability in RFBs.^178^

Choi et al.^179^ studied the impact of membrane thickness on the performance of proton exchange membrane fuel cells (PEMFCs) and proton exchange membrane water electrolyzers (PEMWEs) using four Nafion membranes of different thickness (25, 50, 127, and 183 μm). Their findings demonstrated that both systems are sensitive to variations in membrane thickness, with cell performance improving as membrane thickness decreases. This improvement is attributed to a reduced ohmic resistance, enhanced proton conductivity, promoted water diffusion, enhanced ionic conductivity, and decreased electroosmotic drag. However, the extent to which membrane thickness influences performance differs between the two systems due to variations in operating current densities, water management, and risk of gas crossover. PEMWEs exhibited a higher sensitivity to membrane thickness compared to PEMFCs. This is largely due to their more demanding electrochemical and thermal conditions, such as higher current densities and elevated operating temperatures, both of which accelerate membrane degradation. In contrast, PEC cells typically operate at current densities that are one to 2 orders of magnitude lower than those in electrolyzers.^180^ As a result, PEC systems are generally less sensitive to changes in membrane thickness. Nonetheless, optimizing membrane thickness remains crucial for achieving a balance between high efficiency, durability, acceptable levels of hydrogen crossover, and overall device performance.

In conclusion, finding the right balance between key membrane properties, such as thickness, ion conductivity, membrane resistance, and crossover, is crucial for optimizing performance in various electrochemical applications. These properties must be carefully tailored to meet the specific requirements of the application, as the ideal balance can vary depending on the desired function and the chemical environment in which the membrane operates.

## 6 Conclusions and Future Perspectives

One of the pressing challenges in the field of green hydrogen production is the replacement of halogenated carbon polymers, such as perfluorinated sulfonic acid (PFSA) membranes, which, despite their excellent proton conductivity and chemical stability, pose environmental and economic concerns due to their persistence and high production costs. Thus, in recent years extensive research has been dedicated to the development of advanced IEMs that offer lower costs, ease of synthesis, enhanced thermal and mechanical stability, and environmental sustainability compared to conventional PFSA membranes. This review highlights several promising membrane materials, including aromatic polysulfones and poly­(phenylene oxide), as well as biopolymers such as cellulose and chitosan, which are considered sustainable alternatives for IEM fabrication. Polysulfone-based membranes exhibit excellent mechanical strength, high chemical resistance, and outstanding thermal stability, while PPO-based membranes demonstrate low moisture absorption and high resistance to acidic and basic environments. To optimize the physicochemical properties of AEMs and CEMs for specific applications such as in (photo)­electrochemical cells, various functionalization strategies and fabrication techniques have been explored. Membrane modification can be achieved through the introduction of functional groups such as −SO_3_^–^ or −COO^–^, or via cross-linking with other polymers, enhancing both stability and selectivity. Additionally, surface modification techniques, such as plasma treatment, can improve hydrophilicity, ion transport efficiency, and membrane durability. To align with green chemistry principles, membrane synthesis and fabrication should prioritize sustainable and environmentally friendly methods, incorporating green solvents, catalysts, and minimizing the use of hazardous chemicals, byproducts, and waste generation.

The characterization of IEMs using advanced analytical techniques, such as EIS and AP-HAXPES, provides valuable insights into membrane structure and functionality, aiding in the optimization of ionic conductivity, chemical stability, and electrochemical performance. Furthermore, performance evaluations in PEC devices are essential to assess membrane efficiency, particularly in hydrogen production applications, where oxidation and reduction reactions can be tuned to generate high-value products, such as dihydroxyacetone (DHA) or glyceraldehyde from glycerol oxidation.

Despite significant advancements, further research is required to develop sustainable and high-performance alternatives to fluorinated membranes. A combination of in-depth structural characterization and long-term operational stability studies is crucial for a comprehensive understanding of membrane functionality and degradation mechanisms. Future research should focus on eco-friendly synthesis approaches, industrial scalability, and the development of advanced membrane architectures to drive the progress of next-generation sustainable IEMs.

The integration of artificial intelligence (AI) and machine learning (ML) into membrane research is currently emerging as a transformative approach to accelerate the discovery, characterization, and optimization of next-generation IEMs, particularly those free from halogenated components. Traditional experimental methodologies, while invaluable, often involve time-consuming trial-and-error processes that may not efficiently explore the vast chemical and structural design space required for sustainable membrane development. AI and ML offer the capability to analyze complex data sets, identify patterns, and predict material properties, thereby streamlining the development pipeline.^181,182^ Recent studies have demonstrated the efficacy of ML algorithms in designing fluorine-free copolymers for anion exchange membranes (AEMs), achieving high hydroxide ion conductivity alongside desirable mechanical properties and chemical stability. By training models on existing data sets and employing high-throughput screening, researchers have identified numerous potential candidates that meet or exceed the performance of traditional fluorinated membranes.^183^ These advancements not only reduce environmental impact but also address the economic challenges associated with fluorinated polymers.

Beyond material discovery, AI and ML facilitate the characterization and optimization of membrane properties. Advanced modeling techniques, such as graph-based generative design and molecular dynamics simulations, enable the prediction of key performance metrics, including ion conductivity, water uptake, and mechanical strength.^184^ These predictive models expedite the development process and reduce reliance on resource-intensive experimental procedures. Furthermore, the application of explainable AI (XAI) techniques ensures that the decision-making processes of these models are transparent, fostering trust and facilitating the interpretation of complex data.^185,186^ AI and ML also play a crucial role in understanding and mitigating membrane degradation mechanisms. By analyzing operational data and simulating long-term usage scenarios, ML models can predict degradation pathways and suggest design modifications to enhance membrane longevity. This predictive capability is essential for developing membranes that maintain performance over extended periods, a critical requirement for industrial applications. Looking forward, the synergy between AI/ML and membrane technology holds the promise of revolutionizing green hydrogen production. By enabling the rapid development of eco-friendly, high-performance membranes, these technologies can facilitate the transition away from halogenated polymers, aligning with environmental sustainability goals. Continued interdisciplinary collaboration, coupled with advancements in AI/ML methodologies, will be instrumental in realizing the full potential of this integration, ultimately contributing to the advancement of clean energy solutions.
